# CD44 Targeted Nanomaterials for Treatment of Triple-Negative Breast Cancer

**DOI:** 10.3390/cancers13040898

**Published:** 2021-02-20

**Authors:** Ghazal Nabil, Rami Alzhrani, Hashem O. Alsaab, Mohammed Atef, Samaresh Sau, Arun K. Iyer, Hossny El Banna

**Affiliations:** 1 Department of Pharmacology, Faculty of Veterinary Medicine, Cairo University, Giza 12211, Egypt; gazal_nabil@cu.edu.eg (G.N.); m.Ataf@vet.cu.edu.eg (M.A.); 2 Use-Inspired Biomaterials & Integrated Nano Delivery (U-BiND) Systems Laboratory, Department of Pharmaceutical Sciences, Eugene Applebaum College of Pharmacy and Health Sciences, 259 Mack Ave, Wayne State University, Detroit, MI 48201, USA; r.zhrani@tu.edu.sa (R.A.); h.alsaab@tu.edu.sa (H.O.A.); gi7517@wayne.edu (S.S.); 3 Department of Pharmaceutics and Pharmaceutical Technology, Taif University, P.O. Box 11099, Taif 21944, Egypt; 4 Molecular Imaging Program, Barbara Ann Karmanos Cancer Institute, Wayne State University, School of Medicine, Detroit, MI 48201, USA

**Keywords:** TNBC, theranostic, nanomaterial, momelotinib, JAK/STAT pathway, CARP-1, esterase-responsive nanoparticle, combination therapy

## Abstract

**Simple Summary:**

Triple-negative breast cancer (TNBC) is one of the most challenging tumors with aggressive behavior, low recovery rate, poor prognosis, high metastatic potential, and rapid relapse compared to other breast cancer subtypes. Conventional therapies currently have minimal effect on TNBC; thus, using combination therapies is a valid strategy to enhance drug activity and minimize the overall adverse effect. Therefore, combining drugs with a different mechanism of actions such as apoptosis inducers and JAK/STAT3 inhibitors improved TNBC cell lines killing activity in vitro and in vivo. To further improve the hydrophobic drug activity, CD44 targeted polymeric nanoparticles (CD44-T-PNPs) were utilized by encapsulating hydrophobic drug (CFM-4.16) in CD44-T-PNPs to enhance the drug solubility, tumor accumulation, and most importantly, enhance drug potency. Tagging our PNPs with Hyaluronic acid (HA) enhanced tumor accumulation, reduced off-target distribution, and improved therapeutic efficacy.

**Abstract:**

Identified as the second leading cause of cancer-related deaths among American women after lung cancer, breast cancer of all types has been the focus of numerous research studies. Even though triple-negative breast cancer (TNBC) represents 15–20% of the number of breast cancer cases worldwide, its existing therapeutic options are fairly limited. Due to the pivotal role of the presence/absence of specific receptors to luminal A, luminal B, HER-2+, and TNBC in the molecular classification of breast cancer, the lack of these receptors has accounted for the aforementioned limitation. Thereupon, in an attempt to participate in the ongoing research endeavors to overcome such a limitation, the conducted study adopts a combination strategy as a therapeutic paradigm for TNBC, which has proven notable results with respect to both: improving patient outcomes and survivability rates. The study hinges upon an investigation of a promising NPs platform for CD44 mediated theranostic that can be combined with JAK/STAT inhibitors for the treatment of TNBC. The ability of momelotinib (MMB), which is a JAK/STAT inhibitor, to sensitize the TNBC to apoptosis inducer (CFM-4.16) has been evaluated in MDA-MB-231 and MDA-MB-468. MMB + CFM-4.16 combination with a combination index (CI) ≤0.5, has been selected for in vitro and in vivo studies. MMB has been combined with CD44 directed polymeric nanoparticles (PNPs) loaded with CFM-4.16, namely CD44-T-PNPs, which selectively delivered the payload to CD44 overexpressing TNBC with a significant decrease in cell viability associated with a high dose reduction index (DRI). The mechanism underlying their synergism is based on the simultaneous downregulation of P-STAT3 and the up-regulation of CARP-1, which has induced ROS-dependent apoptosis leading to caspase 3/7 elevation, cell shrinkage, DNA damage, and suppressed migration. CD44-T-PNPs showed a remarkable cellular internalization, demonstrated by uptake of a Rhodamine B dye in vitro and S0456 (NIR dye) in vivo. S0456 was conjugated to PNPs to form CD44-T-PNPs/S0456 that simultaneously delivered CFM-4.16 and S0456 parenterally with selective tumor targeting, prolonged circulation, minimized off-target distribution.

## 1. Introduction

Cancer is known to be the second leading cause of death worldwide after cardiovascular diseases. In 2020, it was estimated that the USA would have approximately 1,806,590 new cancer cases and 606,520 cancer deaths [1]. Since cancer is a global health problem, many efforts are compiled to provide the best possible care to save lives. One of these cancer types is breast cancer, which is considered the second most common cancer diagnosed in women in the USA after lung cancer. Breast cancer afflicts both women and men, but it is far more common in women. Among eight USA women, one develops breast cancer (1:8), and among 883 USA men, one develops breast cancer (1:883) during their lifetime. Breast cancer is molecularly classified based on the presence or absence of specific receptors to luminal A, luminal B, HER-2+, and triple-negative breast cancer (TNBC). TNBC represents 15–20% of whole breast cancer cases. The TNBC is associated with vital organ metastasis, such as lung, bone, liver, pleura, and brain [2], with a median survival period of 9–12 months with conventional chemotherapeutic agents [3]. Owing to the lack of estrogen receptors (ER), progesterone receptor (PR), and HER-2+ receptors, TNBC patients cannot benefit from currently available receptor-mediated systemic therapy such as antiestrogens (tamoxifen) or steroidal and non-steroidal aromatase inhibitors (exemestane & letrozole) and anti-HER2+ (trastuzumab). Moreover, In the last decades, slight therapeutic progress for TNBC has been made, which kept the surgery alone or coupled with chemotherapy, with its side effects, the only available options for TNBC [4,5].

Conventional chemotherapeutic agents with their suboptimal outcomes create an urgent need for developing new approaches to enhance TNBC therapeutic efficacy. In the same vein, combination therapy for TNBC has received significant attention because of the way it enables a simultaneous attack of different aspects of the disease with promising outcomes. Many combination strategies for TNBC are under extensive studies, such as adjuvant (post-operative), neoadjuvant (pre-operative) [6], sequential [7], and simultaneous [8] combination. The reason behind such concern is the capacity of the combination paradigm to refine the conventional chemotherapeutic agents, reduce each drug’s toxicity, overcome chemotherapeutic resistance, and maximize the drug potency compared to a single drug [8].

**Figure 1:**
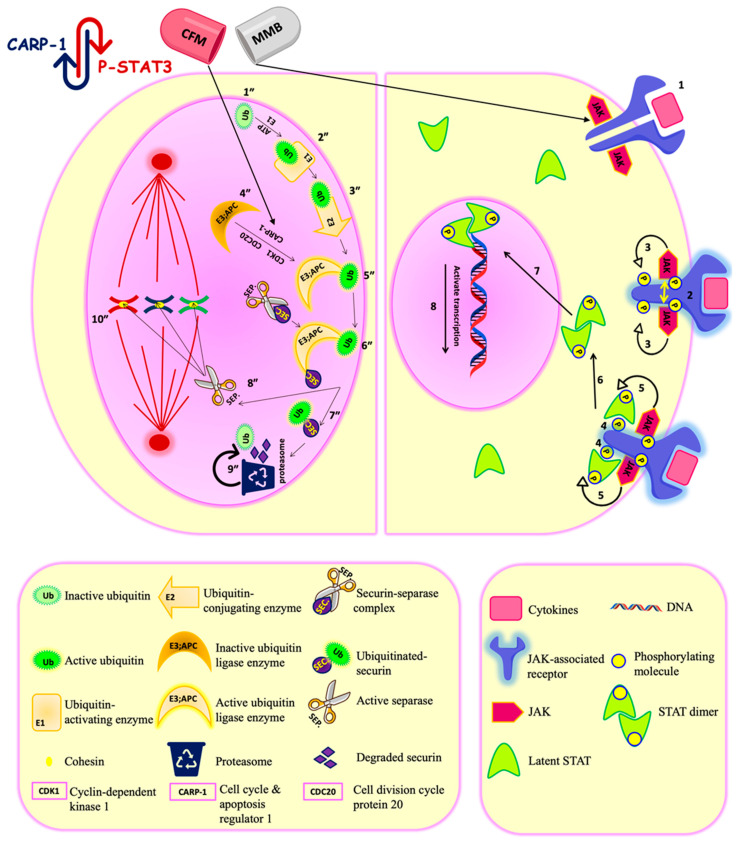
Momelotinib and CFM4.16 synergistic combination. Momelotinib is a JAK-STAT inhibitor. JAK-STAT pathway is a direct signaling pathway transfer the signal from extracellular to nucleus to control the expression of certain genes. The upregulation of the JAK-STAT pathway is the key player in different cancers, and its regulation is under clinical investigation for cancer therapy. The principal components of this pathway are: cytokines-receptor complex, JAK, and STAT proteins. Mechanistically, when the ligand bind to its corresponding JAK-associated receptor (1), the receptors arms are brought into proximity, which enables transphosphorylation between the two JAK molecules (2). The activated phosphorylated JAK subsequently phosphorylates the receptor arms, which is the binding site for the latent transcription factors STAT (3). After the STAT molecules bind to the receptor arms (4), they become ready for phosphorylation by JAK (5). Once phosphorylated, the two STAT monomers dimerize through reciprocal phosphotyrosine-SH2 domain interaction (6). The STAT dimer is an active transcriptional factor that is translocated to the nucleus (7) and binds to a specific DNA sequence in the target gene promoters by DNA-binding domain to control transcription of specific genes (8). Momelotinib antagonizes the ATP binding to JAK1/2, leading to inhibition of the JAK-STAT pathway. CFM4.16 is CARP-1/APC/C interaction inhibitor. APC/C is E3 ubiquitin ligase responsible for tagging cell cycle proteins for proteasomal degradation for the metaphase/ anaphase cell cycle transition. Aberrant APC/C system is associated with cancer progression. Mechanistically, the process starts with latent ubiquitin (Ub) molecules present in the cells (1″), which is activated by Ub-activating enzymes (E1) in an ATP-dependent manner(2″). The activated (Ub)will be transferred to a Ub-conjugating enzyme (E2) (3″), which will conjugate the Ub molecules to activated Ub ligase (E3)/APC/C. The E3/APC/C is under the control of CDK-1(cyclin-dependent kinase-1), CARP-1, and CDC 20 (cell division cycle protein 20).The CDK-1 phosphorylates the APC/C, while CARP-1 binds to the APC2 subunit of APC/C for coactivation. Then the phosphorylated APC/C will bind to CDC 20 to be fully activated (4″). Then The (E2) conjugates the Ub molecules to activated Ub ligase (E3)/(APC/C) (5″). Activated APC/C system ubiquitinates the securin protein (chaperone) to be marked for proteasomal degradation(6″&7″). After the degradation of securin, the separase (separin) will be activated and will break down the cohesin protein between two sister chromatids (8″). Break down of cohesin will lead to the separation of two sister chromatids in anaphase (10″). Thus, APC/C is responsible for maintaining normal chromosome number and genetic stability. Also, APC/C is responsible for turning over S/M cyclins to terminate mitosis. The proteasome catalytic unit will degrade the tagged protein into a small peptide chain and Ub. The Ub will be reused, and the fate of the peptide chain will depend on the cell needs; either it will be repurposed for protein synthesis or energy production (9″). The simultaneous down-regulation of STAT3 and APC/C activation is the underlying mechanism for their synergism.

The JAK-STAT pathway is one of the rapid and direct cascades, whereby the extracellular signals are transferred to the nucleus to control specific gene expression [9]. JAK stands for “Janus Kinase,” while STAT stands for “Signal Transducer and Activator of Transcription.” The relation between the activated JAK/STAT pathway and malignancy development was first brought to attention in the 1990s [10]. Among the seven members of the STAT family, STAT3, particularly phosphorylated by JAK2, is one of the major players that has been detected in many cancers, including the brain, breast, ovarian, pancreatic, prostate, melanoma, squamous cell carcinoma [11], and lung [12]. The cytokines secreted by tumor and tumor microenvironment cells are the critical regulators for the JAK/STAT pathway, which is responsible for cancer development and metastasis. Thus, targeting this pathway has offered a promising approach in controlling solid tumors. Momelotinib is an orally JAK1 and JAK2 inhibitor. It antagonizes ATP binding with JAK1 and JAK2, which leads to inhibition of the JAK2/STAT3 pathway resulting in tumor regression [13], as shown in Figure 1.

Furthermore, the study has developed an apoptosis inducer (CFM-4.16) that stands for CARP-1 functional mimetics (CFMs). CARP-1/CCAR1 (Cell cycle and apoptosis regulator 1) is a peri-nuclear phospho-protein which plays a vital role in regulating cell proliferation and apoptosis pathways. Two E3 ubiquitin ligases govern the cell cycle; APC/C (anaphase-promoting complex/cyclosome) and SCF (Skp1, Cullins, F-box proteins). They tag various regulatory proteins with ubiquitin to be degraded with the proteasome to control many cell functions such as cell cycle, signal transduction, and DNA replication [14,15]. APC/C mediates metaphase/anaphase cell cycle transition and it is under the control of CDK-1(cyclin-dependent kinase-1), CARP-1, and CDC 20 (cell division cycle protein 20). CFM 4.16 is inhibitor of CARP-1-APC/C interaction in APC2 subunit leading to (1) interfere with the cell cycle transition function of APC/C; (2) accumulation of CARP-1. Accumulated CARP-1 induces apoptosis by stimulating tumor suppressors, such as p53, caspase-9, and p38 MAPK, and inhibiting oncogenes, such as c-Met and c-Myc. CARP-1 knocking -down resulted in apoptosis resistance, which indicates the importance of CARP-1 for cell proliferation and apoptosis [16,17], as shown in Figure 1.

Following the recommendation to apply a simultaneous combination therapy in cases of urgent mitigation of tumor burden is required, especially in advanced metastatic cancers, the study has opted for a co-administration MMB and CFM-4.16 for malignant TNBC. In an attempt to surpass the outcome of monotherapy, the employed simultaneous combination paradigm has pivoted upon a repurposing of combined old conventional drugs―reaching the result that: Simultaneous combination overcomes multidrug resistance with increased survivability [8]. 

Due to their illustrated ability to improve chemotherapy safety profile, enhance cancer targetability, allow burst/sustained drug release, and prevent premature drug degradation, nanoparticles (NPs) have been lately extensively investigated—particularly their effectiveness for cancer therapy [18]. One NP cargo could be used for theranostic purpose or a simultaneous combination. The bioavailability of NPs- encapsulated drugs can be improved by anti-fouling or zwitterionic agents, as they will delay NP renal filtration and prevent non-specific accumulation in the reticuloendothelial system (RES) [18]. Many nanoformulations are successfully marketed for cancer therapy as they can overcome the limitation of conventional anticancer therapy such as Doxil^®^, Abraxane^®^, and Genexol-PM^®^ [18]. 

In the study, the employed polymeric NPs (PNPs)—one of multiple NPs platforms which consists of block copolymers, D-alpha-tocopheryl polyethylene glycol succinate (Vitamin E TPGS), and styrene-maleic acid (SMA)— showed improvement in drug solubility and in vitro and in vivo biodistribution. The biocompatibility and degradability of the chosen nanoplatform made it the right candidate for hydrophobic drug delivery. TPGS is an approved FDA delivery carrier due to its inherent preferential features, as well as its ability to inhibit P-glycoprotein (P-gp) associated with multidrug resistance (MDR) [19]. SMA is well-suited for clinical translation due to low cost and ease of processing [20,21]. PNPs surface can be decorated with targeting molecules to increase the bio-affinity to cancerous cells. Hyaluronic acid (HA) is the targeting ligand for a cluster of differentiation-44 (CD44) receptors. CD44 normally expressed in embryonic cells, bone marrow, and connective tissue, CD44 is abnormally extensively expressed in pancreatic, breast, and lung cancers, especially in stem cell subpopulations. CD44 expression indicates poor prognosis, metastasis, EMT mediated chemotherapeutic resistance, and low survivability. For that matter, many contemporary studies support the potential benefits of combining chemo or radiotherapy with CSCs-targeting therapy to overcome tumor resistance and relapse. CD44 in breast cancer is associated with increased (P-gp) and Bcl gene expression responsible for MDR and apoptosis resistance. Based on the clinicopathological impact of CD44 in breast cancer basal type, it is applied as a molecular diagnostic marker, prognostic tool, therapeutic target, targeting ligand-receptor in various stages of clinical development. It is worth noting that whereas CD44 binds to several ligands such as chondroitin, osteopontin, fibronectin, collagen, and serglycin/sulfated proteoglycan, HA remains the specific ligands for CD44 and its all isomers. Moreover, HA is considered the main extracellular matrix component expressed by cancer and stromal cells [22]. HA is widely implemented in cancer therapies due to its intrinisic properties such as biocompatability, biodegradability, safety, non- immungenicity, non-inflammatory, anionic charge, simple linear structure, and ease processing by modifing its funcional groups such as carboxy, hydroxy and N-acetyl groups. Based on the above, tagging our PNPs with HA enhanced tumor accumulation, reduced off-target distribution, and improved therapeutic efficacy.

## 2. Results

### 2.1. The Cytotoxicity of The Individual Drugs and Their Combinations Studies

**Figure 2:**
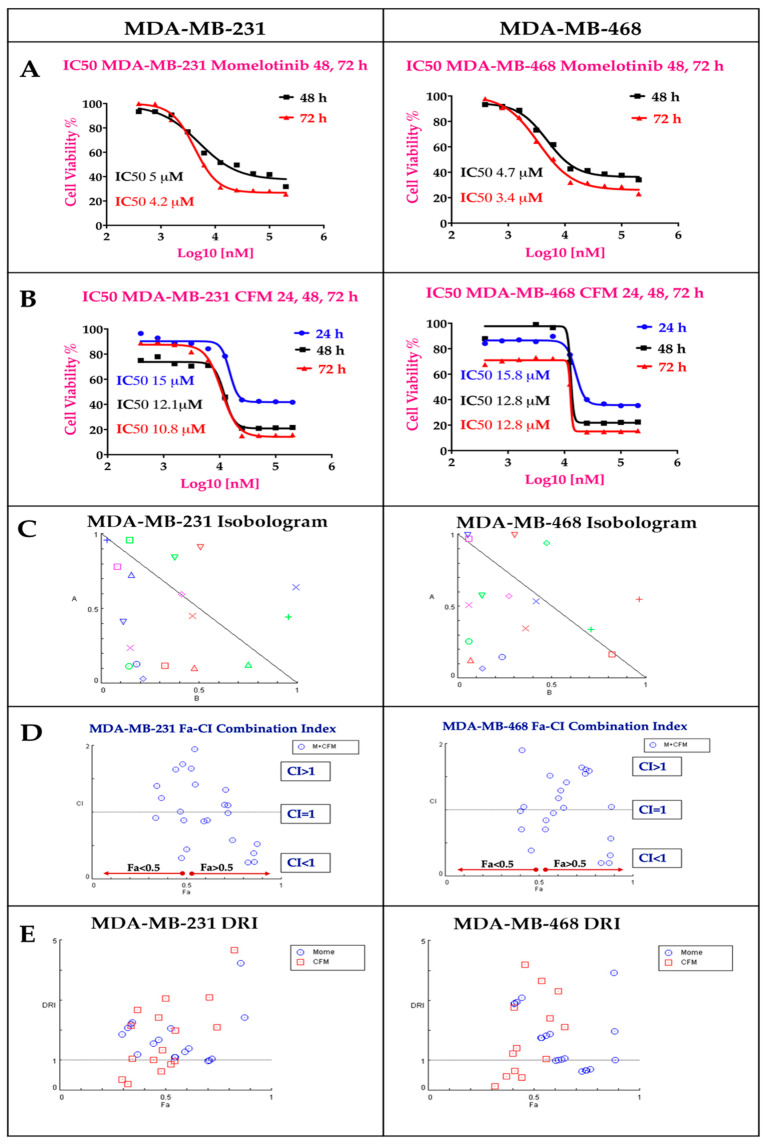
The individual MMB and CFM-4.16 cytotoxicity, Isobologram, Fa-CI index, and Dose Reduction Index (DRI) for the combination of MMB and CFM-4.16 in both MDA-MB-231 and MDA-MB-468 analyzed by COMPUSYN software. (**A**) MMB dose-response curve at 24, 48, 72 h and (**B**) CFM-4.16 dose response curve at 24, 48, 72 h In Isobologram (**C**) the CI < 1, CI = 1, and CI > 1 indicate synergism, additivity, and antagonism, respectively. (**D**) The Fa-CI index of MMB + CFM-4.16 combination at which the synergistic points have CI < 1 with Fa > 0.5. In the DRI (**E**), DRI < 1, DRI = 1, and DRI > 1 indicate unfavorable dose reduction, no dose reduction and favorable dose reduction. Note: MMB means momelotinib.

Both MMB and CFM-4.16 exhibited dose-dependent cytotoxicity with IC50 values of (4.2 & 3.4 µM) and (10.8 & 12.8 µM) in MDA-MB-231 and MDA-MB-468, respectively at 72 h as illustrated in Figure 2A,B. MMB potentiated the cytotoxicity of CFM-4.16 in TNBC cell lines with marked dose reduction and cell whipping, as illustrated in Figure 2 and Figure S1, at which the data is analyzed by COMPUSYN software. The isobolograms in Figure 2C showed that MMB + CFM-4.16 combination had many synergistic points with CI < 1, while others with CI>1 are antagonistic and those with CI = 1 are additive. The MMB + CFM-4.16 showed effective synergism accompanied by high fraction affected (Fa > 0.5) and CI < 1, as shown in Figure 2D. Also, MMB reduces the required concentration of CFM-4.16, as it is evident by (DRI) in Figure 2E. Combination points with DRI > 1 is favorable, DRI = 1 are with no effect, and DRI < 1 are unfavorable. All the CI points of the MMB + CFM-4.16 in both MDA-MB-231 and MDA-MB-468 are present in the Figure S1. MMB + CFM-4.16 combination had a strong synergistic effect (CI ≤ 0.5) on MDA-MB-231 and MDA-MB-468. In MDA-MB-231, 1.5 µM MMB + 12.5 µM CFM-4.16 wipe out 74.4% of cancer cells with CI = 0.58 and DRI = 9.65 and 2.09 for MMB and CFM-4.16, respectively. In MDA-MB-468, the 1.5 µM MMB + 12.5 µM CFM-4.16 wipe out 87.3% of cancer cells with CI = 0.196 and DRI = 7.9 and 14.32 for MMB and CFM-4.16, respectively. 

### 2.2. Polymeric Nanoparticles (PNPs) Formulations

#### 2.2.1. Synthesis of the Non-Targeted SMA-TPGS Carrier and Targeted HA-SMA-TPGS-Carrier

The synthesized non-targeted SMA-TPGS (NT-PNPs) and targeted HA-SMA-TPGS (CD44-T-PNPs) carriers were characterized by FTIR and ^1^H NMR spectroscopy, as shown in (Figures S2 and S3). The reaction between TPGS and SMA generates SMA-TPGS compound, which was confirmed in IR spectra by the presence of four characteristic bands at 3500, 3000,1750, 1250 cm^−1^ due to O-H, C-H of aromatic hydrocarbon, C=O, and C-O-C groups, respectively. ^1^H NMR spectrum of SMA-TPGS shows the characteristic chemical shift at 6.685–7.153 ppm (aromatic H peaks of SMA) and 4.014 ppm (CH2-O- of TPGS). While HA, TPGS, and SMA underwent three pots reaction to produce a product identified as HA-SMA-TPGS. The IR spectra exhibit four bands at 3500, 3100, 1750, 1150, and 1050 cm^−1^ due to OH, C-H of aromatic hydrocarbon, C=O, C-N, and C-O-C groups, respectively. N-H band is overlapped with the O-H band in the IR spectrum. ^1^H NMR spectrum of HA-SMA-TPGS shows the characteristic chemical shift at 6.711–7.2 ppm (aromatic H peaks of SMA), 4.014 ppm (CH2-O- of TPGS), and 4.4–4.6 ppm (hyaluronic acid H in sugar rings). The retention of the characteristic IR and ^1^H NMR peaks of the individual monomers (HA, SMA, TPGS) in the produced SMA-TPGS and HA-SMA-TPGS indicate successful coupling and confirm the formation of the conjugated polymers. The nontargeted and targeted conjugates will be self-assembled into PNPs in the aqueous media due to the presence of hydrophobic SMA polymer and hydrophilic TPGS and HA polymers. The formed PNPs will be water-soluble with a hydrophobic core. The hydrophobic core could be physically or chemically incorporated with hydrophobic drugs for parenteral administration.

#### 2.2.2. Preparation and Characterization of CFM-4.16 Loaded Polymeric Nanoparticles (PNPs)

**Figure 3:**
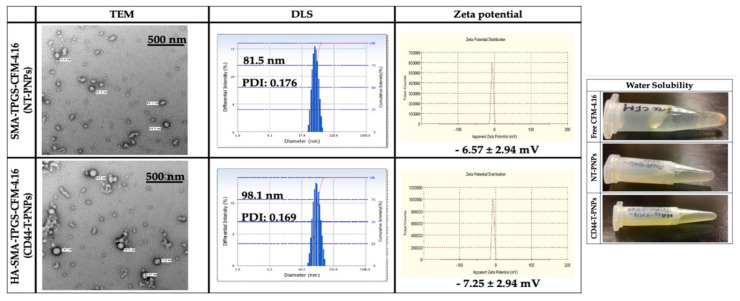
Nanoparticle characterization. Particle characterization including morphology by TEM with 500 nm scale bar, hydrodynamic size by DLS, zeta potential by zeta sizer and water solubility. Both NT-PNPs and CD44-T-PNPs are completely water soluble that enable their parenteral administration compared to non-soluble free CFM-4.16.

The TEM revealed that NT-PNPs and CD44-T-PNPs are spherical with a smooth surface. The DLS average particle size of NT-PNPs was 81.5 nm and of CD44-T-PNPs was 98.1 nm with a narrow polydispersity index (PDI) 0.176 and 0.169, respectively, consistent with TEM results. The surface charge of NT-PNPs was 6.57 ± 2.94 mV and of CD44-T-PNPs was -7.25 ± 2.94, as shown in (Figure 3). CD44-T-PNPs had a larger particle size and negative zeta potential, attributed to wrapping the PNPs surface with HA. The water solubility of both formulations favors their parenteral administration. The loading contents (LC%), encapsulation efficiency (EE%), and yield% of NT-PNPs and CD44-T-PNPs are presented in (Table 1) as mean ± SD, *n* = 3. 

#### 2.2.3. CD44 Targeted Polymeric NPs Mediates Cellular Uptake via CD-44 Overexpression on TNBC Cell Lines

**Figure 4:**
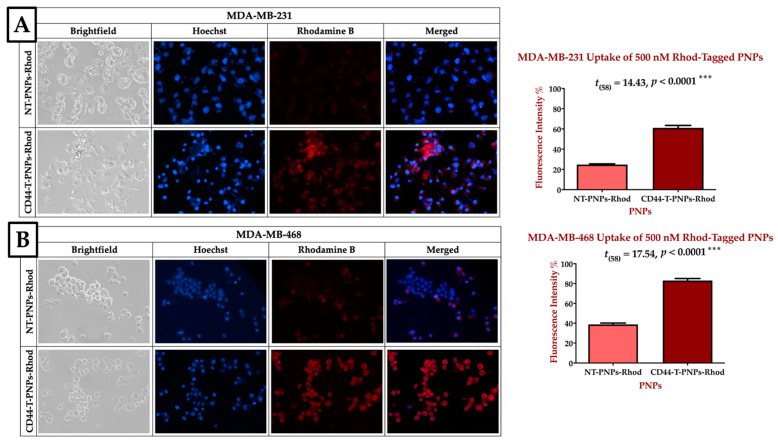
CD44 receptor-mediated selective delivery of CD44-T-PNPs in TNBC cell lines. MDA-MB-231 (**A**) and MDA-MB-468 (**B**) were selectively uptake CD44-T-PNPs/Rhodamine in comparison to NT-PNPs/Rhodamine after treatment with 500 nM for 1.5 h. The intensity was measured by Zen 2012 (blue edition), and significance was calculated by *t*-test in Prism. The data are present as a mean ± SEM. Magnification is 40×.

CD44-T-PNPs/Rhodamine had better tumor accumulation than NT-PNPs/ Rhode in CD44 overexpressing MDA-MB-231 and MDA-MB-468 cell lines, as shown in Figure 4. Blue fluorescence refers to Hoechst stained nuclei, while red fluorescence refers to the Rhodamine B uptake signal. In MDA-MB-231, CD44-T-PNPs/Rhod had 2.5 folds better tumor accumulation compared to NT-PNPs/Rhod, as shown in Figure 4A. While in MDA-MB-468, the CD44-T-PNPs had 2.1 folds better tumor accumulation compared to NT-PNPs, as shown in Figure 4B. Higher cellular uptake of CD44-T-PNPs affirmed in both cell lines is probably due to receptor-mediated endocytosis followed by HA/CD44 interaction. CD44-targeted nanomaterials could be a useful tool for selective cytotoxicity in TNBC.

#### 2.2.4. CD44-Targeted PNPs Increases Cytotoxicity Against TNBC Cell Lines 

**Figure 5:**
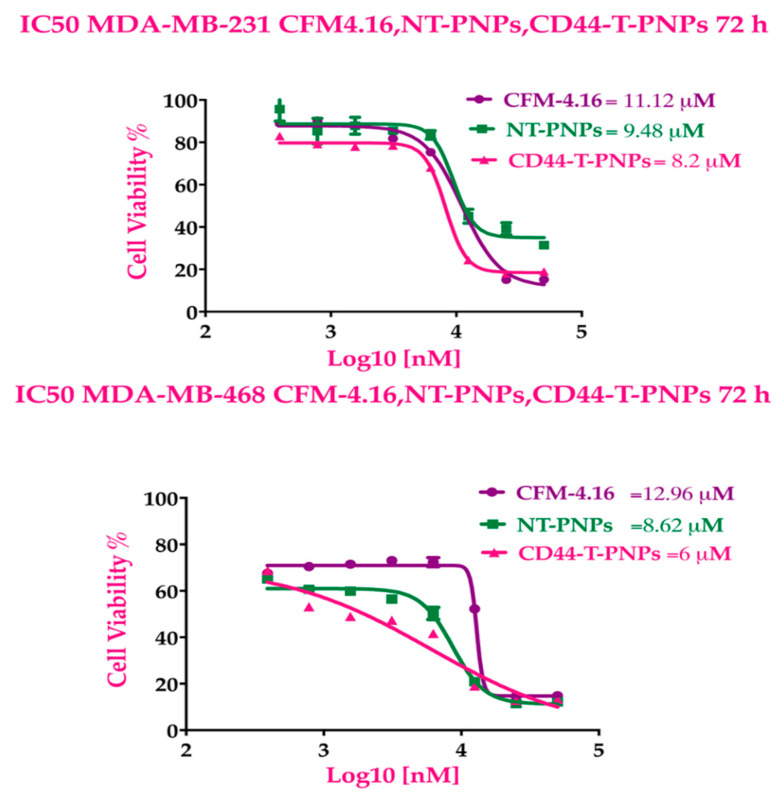
CFM-4.16 formulations cytotoxicity. The dose response curves of CFM-4.16, NT-PNPs and CD44-T-PNPs were plotted for MDA-MB-231 and MDA-MB-468 at 72 h Data represent mean ± SD, *n* = 8.

CD44-T-PNPs showed more cytotoxic effect, followed by NT-PNPs compared to free CFM-4.16. The dose-response curve in Figure 5 indicates that NT-PNPs decreased the IC_50_ by 1.17- and 1.5-fold in MDA-MB-231 and MDA-MB-468, respectively, while CD44-T-PNPs reduced IC_50_ by 1.35- and 2.16-fold in MDA-MB-231 and MDA-MB-468, respectively. The potency of the PNPs may be attributed to the stabilizing and controlled release effect of SMA and the P-gp inhibition by vitamin E-TPGS. The HA/CD44 mediated endocytosis potentiates CD44-T-PNPs cytotoxicity.

### 2.3. Momelotinib + CFM-4.16 Combination Studies and the Cause of Synergism 

#### 2.3.1. Targeted PNPs Combination Has Exhibited Remarkable Anticancer Activity Compared to Free Drugs Against TNBC Cell Lines

**Figure 6:**
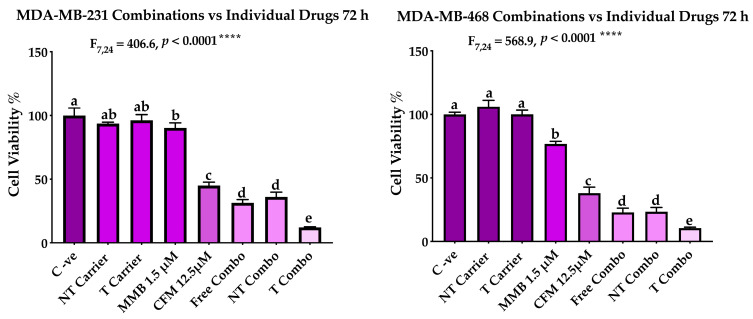
The performance of MMB + CFM-4.16 combinations (free combo, NT combo, and T combo) vs. the individual free drugs. Note: T combo (CD44-T-PNPs + MMB) and NT combo (NT-PNPs + MMB).

**Figure 7:**
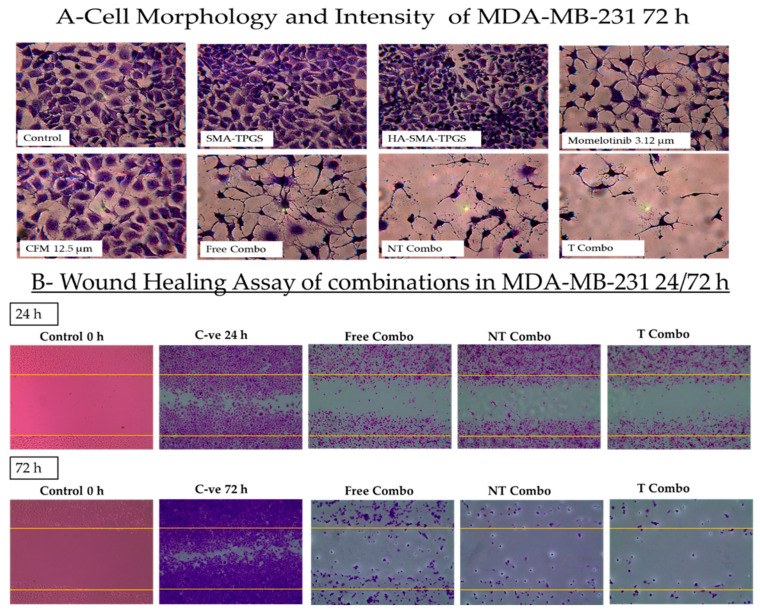
The impact of the combination on the cell morphology and wound healing. The morphological changes (**A**) and the ability of the cells to proliferate and migrate to fill the induced gap in MDA-MB-231 (**B**) is illustrated by crystal violet stain. For the wound healing, the plates were treated as follows; negative control at 0, 24, 72 h, free combo, NT combo, and T combo at 24 and 72 h Note: T combo (CD44-T-PNPs + MMB),NT combo (NT-PNPs + MMB) and free combo (CFM-4.16 + MMB) where MMB means momelotinib. Images were taken at 10× magnification power.

The MMB + CFM-4.16 combination had a more cytotoxic effect than individual MMB and CFM-4.16, as confirmed Figure 5. The cells viability percentage in MDA-MB-231 was 31.3%, 90.2%, and 44.9% for MMB + CFM-4.16, MMB and CFM-4.16, respectively while viability% in MDA-MB-468 was 22.7%, 76.7%, and 37.9% for MMB + CFM-4.16, MMB, and CFM-4.16, respectively, as shown in Figure 6. Interestingly, T combo (CD44-T-PNPs +MMB) had a significant potent cytotoxic effect compared to the free combo, as shown in Figure 6. T combo decreased the cells viability percentage by 2.5 and 2.16 folds in MDA-MB-231 and MDA-MB-461, respectively, compared to the free combo (MMB + CFM-4.16). Moreover, the results illustrated in Figure 7 show a decrease in the proliferation capacity, increase in the cellular detachment, and significant change in the cellular morphology in the combination wells compared to control negative. In combinations wells, especially the targeted one, the cells appear as a star shape with tapered ends with very low density. The co-treatment of MMB + CFM4.16 either in free or in PNPs form inhibited cell migration and wound closure at both 24 and 72 h.

#### 2.3.2. Combination Therapy Has Synergistic Effect Due to ROS Generation, Elevated Caspase 3/7Activity, and Downregulation of P-STAT3

**Figure 8:**
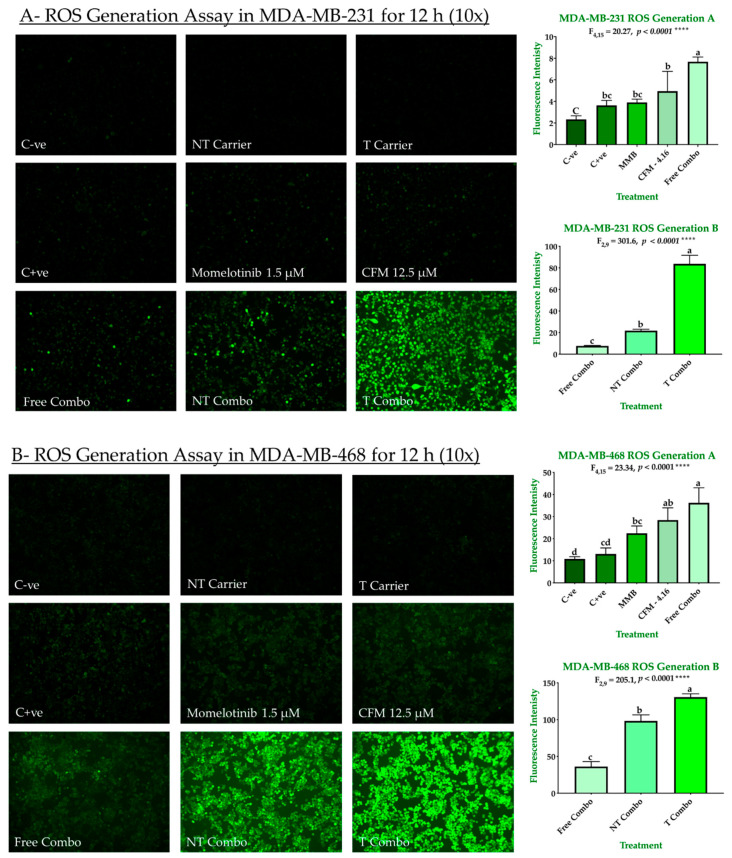
ROS generation in TNBC cell lines. The ability of MMB + CFM-4.16 combinations to promote antitumorigenic signaling and trigger oxidative stress-induced cancer apoptosis was evaluated in MDA-MB-231 (**A**) and MDA-MB-468 (**B**) by fluorescence H2DCFDA dye. The cells were treated for 12 h with MMB, CFM-4.16, free combo, NT-combo, T-combo, and equivalent doses of NT-carrier and T-carrier. H2O2 was used as a positive control. Then the media was removed, and the cells were stained for 30 min with non-fluorescent dye 2′,7′-dichlorodihydrofluorescein Diacetate (DCFH-DA) which is oxidized by the intracellular ROS to 2′,7′-dichlorofluorescein (DCF). The wells were imaged by with (EVOS FL Auto, Life Technologies) microscope with 10 s elapse time and 10× magnification. The intensity was measured by Zen 2012 (blue edition). The data are presented as a mean ± SEM; the means were compared by analysis of variant, post hoced with Bonferoni, different letters indicate significant difference. Note: T combo (CD44-T-PNPs + MMB) and NT combo (NT-PNPs + MMB) and free combo (CFM-4.16 + MMB) where MMB means momelotinib. 10× magnification was used.

The ability of our combinations to induce ROS-dependent apoptosis is shown in Figure 8A,B. Individually, drugs exhibited a slight increase in ROS production in both MDA-MB-231 and MDA-MB-468. In MDA-MB-231, the ROS generation was increased by 1.55, 1.67, 2.12, 3.28, 9.32, and 35.82 for H_2_O_2_, MMB, CFM-4.16, free combo, NT combo, and T combo, respectively compared to negative control. In MDA-MB-468, the ROS generation was increased by 1.2, 2.06, 2.62, 3.33, 9.04, and 12.02 for H_2_O_2_, MMB, CFM-4.16, free combo, NT combo, and T combo, respectively compared to negative control. The NT and T carriers had a slight attenuation in ROS production due to vitamin E’s antioxidant effect in their content [23]. These results suggested that MMB + CFM-4.16 synergistic effect due to the increase in ROS generation. Moreover, T combo, with its unprecedented ROS production, alter the redox environment promoting oxidative stress-induced cancer cell death.

**Figure 9:**
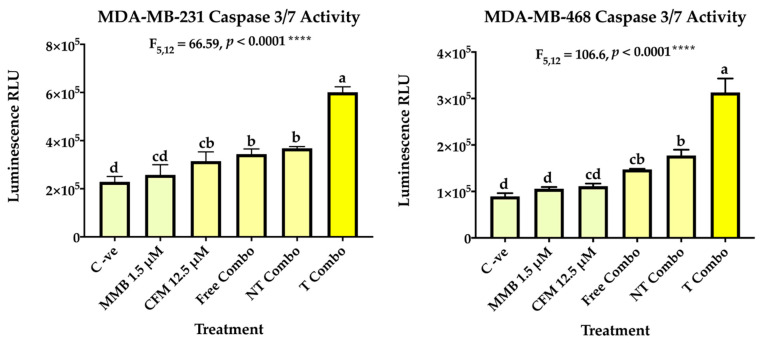
Caspase 3/7 activity. The ability of MMB + CFM-4.16 combinations to induce apoptosis was evaluated in MDA-MB-231 and MDA-MB-468 by Caspase-Glo 3/7 kit. At which, the cells were treated with MMB, CFM-4.16, free combo, NT-combo, and T-combo). The luminescence intensity is directly proportional to the amount of caspase activity present. The data are presented as a mean ± SEM (*n* = 3), the means were compared by analysis of variant, post hoced with Tukey, different letters indicate significant difference. Note: T combo (CD44-T-PNPs + MMB) and NT combo (NT-PNPs + MMB) and free combo (CFM-4.16 + MMB) where MMB means momelotinib.

In addition, caspase 3 and caspase 7 which are crucial for the execution phase of cellular apoptosis have been also investigated. Caspase 3 activation is a conclusive marker for the irreversible commitment of cellular apoptosis. The results have revealed enhancement of caspase 3/7 activity in the free combo compared to the individual drugs and control groups in both cell lines. In MDA-MB-231, caspase activity increased by 1.13 for MMB, 1.39 for CFM-4.16, 1.52 for the free combo, and 1.62 for NT combo compared to negative control cells. In MDA-MB-468, caspase activity increased by 1.18 for MMB, 1.25 for CFM-4.16, 1.67 for the free combo, and 2.01 for NT combo compared to negative control cells. Interestingly, the T combo markedly stimulated caspase 3/7 activity over the free combo; by 1.76 in MDA-MB-231 and 2.14 folds MDA-MB-468, as shown in Figure 9.

**Figure 10:**
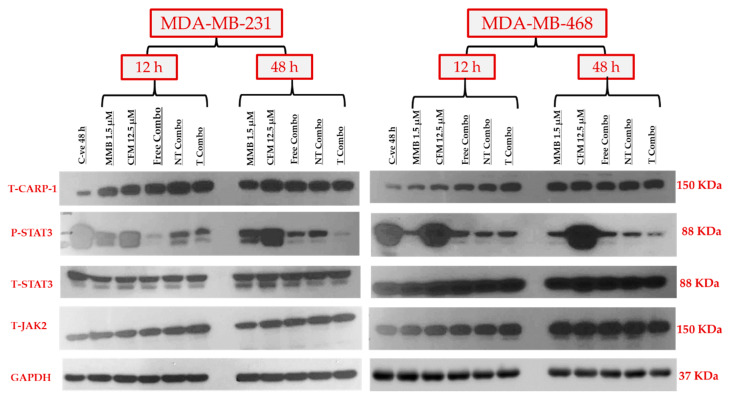
The molecular mechanism of the synergistic anticancer activity of MMB + CFM-4.16 combinations. Western blotting of the oncogenic P-STAT3, tumor suppressor CARP-1, T-STAT3, T-JAK2, and GAPDH for the individual drugs and MMB + CFM-4.16 combinations in both MDA-MB-231 and MDA-MB-468 at 12 and 48 h. MMB + CFM-4.16 combinations showed simultaneous downregulation of P-STAT3 and up-regulation of the CARP-1 compared to the control and individual drugs at both 12 and 48 h in both cell lines. The combinations, especially the T combo, were able to upregulate CARP-1 in the first 12 h compared to individual CFM-4.16 in both cell lines. The potency of NT combo and T combo was owing to their sustained release and prolonged effect on suppressing P-STAT3 both cell lines. Even the free combo was able to completely wipe out P-STAT3 in the first 12 h in MDA-MB-231, yet, the impact was short-term effect as there was little upregulation of P-STAT3 at 48 h unlike the NT and T combo which had stable and ascending cumulative effect. There was no change in the T-SAT3 and T-JAK2 in both cell lines. GAPDH was used as an internal control of protein loading. Note: T combo (CD44-T-PNPs + MMB), NT combo (NT-PNPs + MMB) and free combo (CFM-4.16 + MMB) where MMB means momelotinib. The densitometry and uncropped blots are shown in Figure S5.

The synergistic effect of MMB+ CFM-4.16 combinations was studied by exploring the expression level of the targeted proteins, including P-STAT3, CARP-1, T-STAT3, and T-JAK2, as shown in Figure 10. At the same time, GAPDH has been simultaneously employed as an internal control. The combination synergistic effect is based on the concurrent upregulation of tumor-suppressor CARP-1 and downregulation of the tumorigenic P-STAT3 signaling pathways. Free combo, NT-combo, and T- combo have had concomitant stimulation of CARP-1 and inhibition of P-STAT3 in MDA-MB-231 and MDA-MB-468 at 12 and 48 h compared to control and individual drugs. In the first 12 h, all combinations were able to increase CARP-1 expression compared to standard free CFM-4.16 in both cell lines. NT combo and T combo had sustained prolonged effect compared to free combo as made evident by the expression level of P-STAT3 in MDA-MBA-231 and MDA-MB-468 where free combo wiped out P-STAT3 at 12 h, but the effect slightly retreated at 48 h. On the other hand, the NT combo and T combo showed a significant, sustained ascending cumulative wiping out of P-STAT3 on both cell lines till 48 h. Momelotinib alone was able to increase stress marker CARP-1 in both cell lines in both time points compared to control, whereas CFM-4.16 alone did not affect P-STAT3 in both cell lines at both time points.

### 2.4. Animal Studies

#### 2.4.1. CD44 Receptors Are Overexpressed in Tumors of TNBC-Bearing Mice Model 

**Figure 11:**
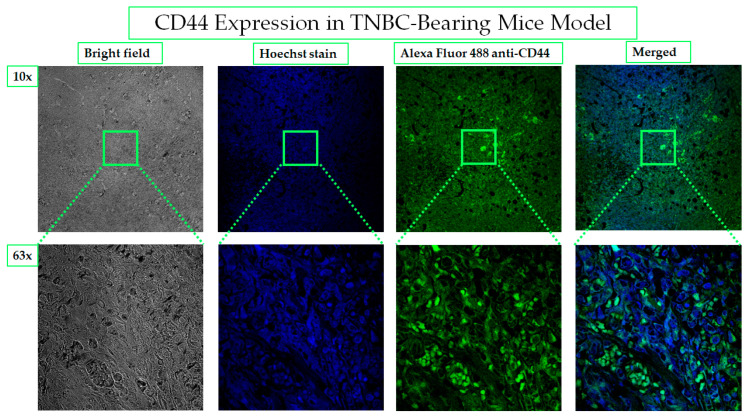
CD44 overexpression in TNBC- bearing mice model. The expression of CD44 in MDA-MB-231- induced TNBC in mice is considered the basis for the selection of HA as a targeting ligand for PNPs. Alexa Fluor 488 conjugated anti-CD44 antibody was used to demonstrate the homogenous distribution of CD44 receptors on the in vivo tumor. The images were taken by a confocal microscope with 10× and 63× magnification power.

The expression of CD44 in ectopic tumor xenograft collected from the TNBC-bearing mice model was investigated by immunohistochemistry. The intense bright green fluorescence indicates the high expression level of CD44, as shown in Figure 11. Molecular characterization leads to the discovery of biomarkers and targeted therapy, which is the basis of personalized medicine. The association of CD44 with tumorigenesis induction, poor prognosis, aggressiveness, relapse, and chemotherapeutic resistance of TNBC has been the underlying rationale behind the study’s choice of CD44 as an excellent biomarker for site-specific payload delivery to TNBC.

#### 2.4.2. NIR Imaging and Biodistribution and Inducible DNA-DSBs

**Figure 12:**
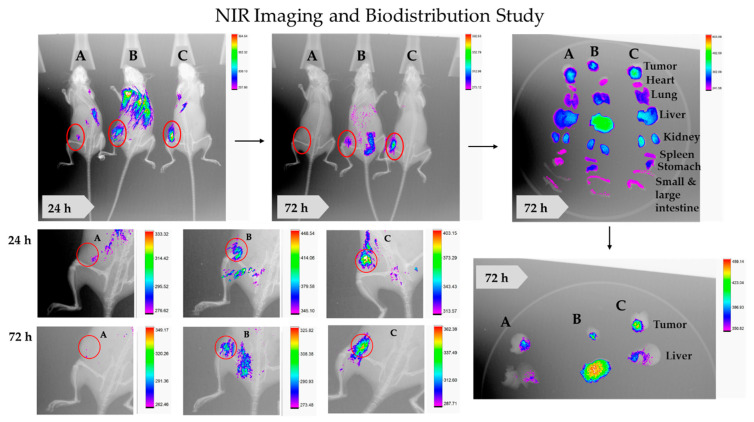
Theranostic NT-PNPs/S0456 and CD44-T-PNPs/S0456 in TNBC-bearing mice model. Free S0456 (**A**), NT-PNPs/S0456 (**B**), and CD44-T-PNPs/S0456 (**C**) were injected via tail-vein into TNBC- bearing mice. The targeted theranostic PNPs showed highly selective tumor uptake, favorable tumor-selective bio-distribution, and payload sustained-release (**C**), Whereas non-targeted theranostic PNPs have minimal tumor uptake with non-specific organ bio-distribution (**B**). The free dye was rapidly eliminated with neglectable tumor accumulation (**C**). Whole-body fluorescence and X-ray merged images were acquired at 24 and 72 h post-injection.

**Figure 13:**
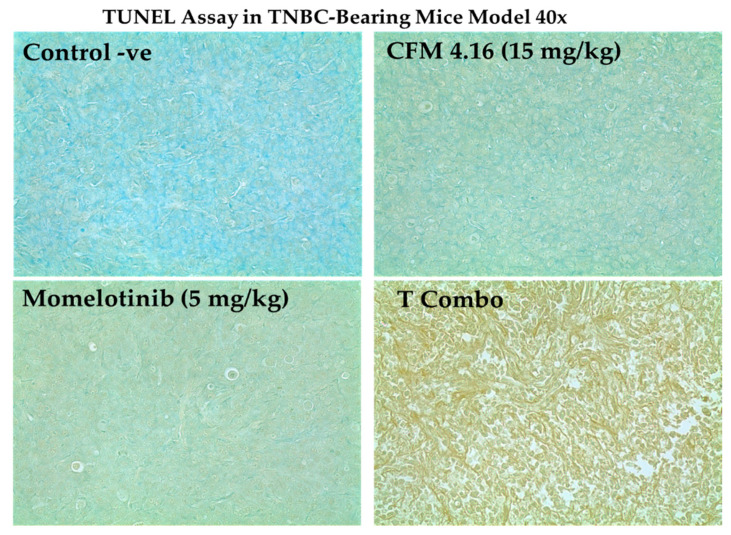
The capability of T combo to enhance the onset of apoptosis in TNBC- bearing mice by CD44. HA mediated endocytosis. T combo was able to drive the tumor to the late-stage apoptosis, which is based on generating multiple DNA double-strand breaks (DSBs) with accessible 3’-hydroxyl (3’-OH) groups that were detected by TUNEL. Elevated apoptosis is indicated by brownish discoloration or dark brown spots. Note: T combo (CD44-T-PNPs + MMB) where MMB means momelotinib. Magnification is 40×.

The theranostic PNPs is an emerging aspect of a precise medicine. It consists of targeting ligand, therapeutic agents, and imaging agents. Sufficiently accumulated in the tumor by enhanced permeability and retention (EPR) and receptor-mediated endocytosis, theranostic NPs is helpful for early diagnosis, image-guided surgery, and tracking drug distribution, accumulation, sustained release, and efficacy. Being less toxic and cost-effective, NIR imaging in TNBC-bearing mice has been opted for by the study. The results have illustrated significant tumor homing of CD44-T-PNPs/S0456, followed by NT-PNPs/S0456, compared to control (free S0456) at both 24 and 72 h, as shown in Figure 12. In addition, the CD44-T-PNPs/S0456 exhibits no off-target accumulation, especially in the liver, compared to NT-PNPs/S0456 at both 24 and 72 h as shown in whole-body and dissected organ imaging Figure 12. The high intensity of the CD44-T-PNPs/S0456 group in the kidney at 72 h has revealed the following (1) hydrophilic nature of the formulation enabled its renal clearance; (2) the formulation had prolonged sustainable property till 72 h; (3) renal mediated excretion reduces liver toxicity; (4) HA surface coating reduced NPs immunogenicity and elimination by RES. The selective homing of CD44 targeted PNPs profoundly support the rational application and clinical translation of it to the theranostic platform of metastatic TNBC. To support the therapeutic efficacy, a TUNEL assay was performed— indicating the results that: the T combo was significantly able to push the tumor cells to late-stage apoptosis when compared to individual drugs, as shown in Figure 13.

## 3. Discussion

TNBC is one of the most challenging tumors with an aggressive behavior, low recovery rate, poor prognosis, high metastatic potential, and rapid relapse compared to other breast cancer subtypes. TNBC abbreviation derived from the deprivation of three types of receptors; estrogen receptors (ER), progesterone receptor (PR), and human epidermal growth factor (HER2) [24]. The discovery of these biomarkers by Perou 2000 played an essential role in the development of targeted, personalized medicine. Due to the lack of these receptors, TNBC patients can not benefit from currently available receptor-targeted systemic therapy, making surgery and chemotherapy the only available option. 

However, traditional chemotherapeutic agents’ application has not been without potential side effects, suboptimal outcomes, and tumor resistance development. In addition, even though most of the chemotherapeutic agents have the capacity to attack fast-growing cancerous cells, they wipe out fast-growing healthy cells too, such as: bone marrow cells, hair follicle cells, and cells lining the gastrointestinal tract (GIT) [25,26,27]. In the same vein, despite the fact that most chemotherapeutic agents can trim tumor growth, its effect is not long-lasting and is followed by rapid proliferation and invasion, which is the reason for the development of chemotherapeutic resistance. Drug resistance is considered the main obstacle that breast cancer patients have to confront and is responsible for chemotherapeutic failure. The hurdle of drug resistance could be innate, acquired, or cross-resistance/MDR with different underlying mechanisms such as drug sequestration by P-gp, proliferation potential of mutated cancer stem cells (CSCs), altering drug target, modification of DNA repair strategies, altering drug detoxification, and invalid apoptotic regulators such as p53 [28,29]. There upon appears the urgent need for new approaches such as immunotherapeutic approach [30], new agents that do not exhibit cross-resistance such as ixabepilone [28], targeting cancer stem cells, and unique combination strategies.

The current study establishes a novel *combination strategy* based on the sensitization of TNBC to CFM-4.16 by MMB, which has proven an unequivocally capacity to overcome single-agent prone -therapeutic resistance, reduce systemic toxicity, and enhance the therapeutic index. Moreover, CFM-4.16 water solubility was enhanced by SMA-TPGS carrier as well as cell uptake using CD44 targeting ligand in vitro and in vivo. It is worth noting that the possibility of using this cargo for the theranostic purpose has been likewise investigated. 

PNPs were first nominated for cancer therapy in the early 1980s. PNPs able to in-crease the water solubility of hydrophobic drugs as they consist of a hydrophilic shell, which interacts with the external aqueous media, and a hydrophobic core, which acts as a depository for hydrophobic drugs [31]. This is in addition to their ability to enhance drug retention in tumor tissue by EPR effect as well as prolonging plasma half-lives by escaping renal elimination [18,32]. One of the polymeric micelles that have been FDA approved for breast cancer is Genexol-PM^®^ [33]. The study has opted for a carrier, which is a block copolymer consisting of SMA and Vitamin E-TPGS, decorated by HA as a targeting ligand. Established as biologically safe and immunostimulant [34,35] SMA was clinically approved for the treatment of hepatoma in Japan in 1993 [36,37]. Having a high glass transition temperature, it increases NPs stability and controls the drug release. Moreover, the carboxyl group of maleic acid on SMA’s hydrophilic surface enables surface modification by conjugation to the targeting ligand, such as HA [20,21,38]. Vitamin E-TPGS is FDA approved drug adjuvant which is widely used as a pharmaceutical emulsifier, stabilizer, and permeation and bioavailability enhancer of hydrophobic drugs with a potent P-gp inhibition and apoptosis induction [19,39,40]. 

PNPs is a promising drug delivery to overcome poor solubility, limited selectivity, and systemic cytotoxicity. It is well documented that PNPs accumulate in the tumor site in a high concentration by utilizing EPR. The main challenge against passively delivered PNPs is that angiogenesis is not uniformly distributed throughout the tumor leading to a disproportional distribution of NPs by EPR [41]. The active targeting based on the microenvironmental difference between cancer and healthy cells is crucial to add selectivity to EPR and overcome its limitations. One such difference is the expression levels of CD44 [42]. CD44 is a cell surface glycoprotein that is overexpressed and intensively involved in TNBC carcinogenesis [22,43]. Basal epithelial, basal mesenchymal TNBC, and CSCs are enriched with the CD44 receptor [44]. Therapeutic failure and cancer relapse are mainly due to the inability to eradicate CSCs [13,45]. Targeting of cancer cells and CSCs will effectively reduce tumor burden and relapse. CD44 has a tremendous binding affinity to HA, which attracts our attention to develop HA-based NPs leading to an increase in the affinity of NPs to cancer cells and CSCs and selective toxicity due to HA-CD44 receptor-mediated endocytosis [42,43]. In our study, HA-PNPs have proved their significance in developing CD44-T-PNPs with high cellular uptake in vitro, preferential tumor accumulation in vivo, and theranostic potential by enveloping both CFM 4.16 and S0456. That came in agreement with HA-SMA-NMS that effectively delivered CDF to the aggressive CD44+ stem-like pancreatic cancer cells [46] and HA-TPGS-DOX that increased doxorubicin cytotoxicity in MCF-7/ADR [47]. Adding vitamin E-TPGS improved Genexol-PM uptake and PTX cytotoxicity due to the enhancement of membrane fluidity and MDR inhibition; the IC_50_ was reduced by 4.4 folds compared to Genexol-PM [40].

Many physiochemical characters of NPs affect their cellular interaction, such as shape, size, surface charge, and hydrophobicity. The study has opted for spherical with a smooth surface, <100 nm, slightly anionic, and water-soluble, which have proven ideal for use in the drug delivery system (DDS). Most of the developed NPs applied for DDS are spherical due to their being easy manufacting.. Even many studies showed that rod or disc NPs have a more favorable effect than spherical, there are contradictory studies [48]. This contradiction is owing to differences in the material composite, tested cell lines, and analyzing techniques [49,50,51]. Another reason why spherical-shaped NPs have been preferred is their lower reactivity and toxicity than fiber-shaped NPs [52]. In addition, whereas the non-spherical ones tumble with the flow, spherical shaped NPs are known for their ease of motion [53]. Furthermore, PNPs’ smooth surface have the ability to reduce the phagocytosis process [54] and deposition rate [55]. The size of NPs is a determent factor for its clinical application. Our PNPs are able to escape glomerular filtration (<5 nm) and trapping by RES (>150 nm) [56]. Also, it is in the favorable size range for cellular uptake and extravasation via EPR. As the cut-off size of the endothelial gaps in tumor blood vessels ranges from 200 nm to 1.2 μm based on tumor type; therefore, NPs ≤ 200 nm are widely used for passive and active targeting of the tumor [57]. Controlling NPs size is critical for reducing genotoxicity (∼10 nm) and cytotoxicity to healthy cells [58]. NPs surface charge plays a vital role in drug loading, circulation time, cellular uptake, cellular cytotoxicity, NPs stability, and clearance by RES. The cationic NPs have many safety concerns, as they are strongly attracted to the negatively charged cell membranes leading to destabilization of cell membranes, leakage of cytoplasm, and, subsequently, cell lysis. Damage includes the endothelial lining of blood vessels, RBCs, and healthy cells. Elimination of NPs by RES based on their zeta potential is a controversial issue. It is generally accepted that neutral or slightly anionic NPs are preferred for their safe parenteral administration, lower systemic toxicity, higher tumor accumulation, prolonged circulatory lifetime, and less off-target uptake [53,56,59,60]. Our hydrophilic PNPs are far more vulnerable to immune detection as the hydrophilic NPs repel opsonions that reduce their recognition by mononuclear phagocyte system (MPS) and increase their circulatory time [49,61].

NT-PNPs and CD44-T-PNPs have an adequate LC% with respect to the polymer amount compared to other polymeric NPs preparation [62]. As it is well established that TNBC has tremendous esterase and hyaluronidase activity [63,64]. Both PNPs are esterase-responsive while only CD44-T-PNPs are additionally hyalu-ronidase-responsive. The carbonic ester bonds and the glycosidic bonds in the NPs will be disassembled by intracellular esterase and hyaluronidase, followed by releasing the antineoplastic agent. This will increase tumor specificity, reduce off-target toxicity, prevent premature drug release and circulation stability [63]. Our PNPs physicochemical properties are consistent with the marketed Genexol-PM^®^ that had a smooth spherical shape, −4.36 mv,16.67% LC, and two folds reduction in IC_50_ compared to free PTX in A549 [40].

The current study has illustrated a promising synergism supported by the in vitro and in vivo anticancer activity. The co-treatment of momelotinib (MMB) and CFM-4.16 increases the downregulation of P-STAT3 accompanied by the upregulation of CARP-1, especially in T-combo. STAT3, particularly phosphorylated by JAK2, is one of breast cancer clinical significance [11]. STAT3 enhances cell proliferation by activating cyclin-dependent kinases (CDKs) by upregulation of cyclin D2 and downregulation of p21. STAT3 induces transcription of hypoxia-inducible factor (HIF-1a). STAT3/HIF-1a axis plays a crucial role in adapting tumor cells to the hypoxic environment associated with cancer progression. Also, STAT3 leads to overexpression of VEGF responsible for angiogenesis. Tumor invasion and metastasis are under the regulation of STAT3 by different mechanisms such as; induction of transcription of matrix-degrading enzymes such as matrix metalloproteinase and activation of epithelial to mesenchymal transition (EMT). STAT3 inhibition, either by knocking down or by pharmacological inhibitors, was found to suppress tumor invasion and metastasis in vivo and in vitro [65]. Inhibition of the JAK/STAT pathway has increased the sensitivity of resistant breast cancer cells to doxorubicin [66]. Worth emphasizing is, JAK2 /STAT3 is a prerequisite for the maintenance and proliferation of CSC of breast cancer and developing of chemo- and radio-resistance [13,67]. So, the JAK2/STAT3 in CSC is a potential target for developing a successful strategy to improve breast cancer patients’ therapeutic outcomes.

The study has revealed that the combination of MMB and CFM-4.16 has induced apoptosis via JAK2/STAT3 inhibition- mediated ROS generation. Recently it has been reported that P-STAT3 is inversely related to ROS production [68,69,70]. ROS is regarded as a double-edged sword in cancer cells as a slight increase of ROS leads to cancer initiation and progression, while high levels of ROS induce cell senescence. It is known that the level of cancer intrinsic ROS is relatively higher than that of normal cells; thus, increasing the ROS production by chemotherapy will effectively eradicate cancer cells, but it will be inadequate to trigger apoptosis in healthy cells with a low level of intrinsic ROS [71]. Redox imbalance induces apoptosis by disturbing mitochondrial membrane potential, enhanced mitochondrial membrane permeability to pro-apoptotic proteins, including cytochrome c. In the cytosol, cytochrome c binds to Apaf-1 to form an apoptosome, which in turn activates caspase-9. Overactivation of caspase-9, in addition to caspase-8, p38 MAPK, upregulation of CARP-1, and PARP cleavage, took place by CFM-4.16 [16,17]. Taken together, activate the caspase 3/7 cascade pathway that was confirmed in our results leading to DNA damage, cell shrinkage, and cellular detachment [72], which were proved by TUNEL, cellular viability, and morphology (Summarized in Figure S4).

Optical imaging by targeted NIR dye has been evolved to enable observation of cancer burden and progression under various therapeutic strategies and stages and rapid monitoring of molecular events occurring within cells. Rapid assessment of the therapeutic efficacy in vivo is highly needed. As in relatively slow-growing models, the caliper measurements are unable to detect the difference for several days. Also, in orthotopic, metastatic, and systemic models, the longitudinal measurements of tumor burden are not possible. In our attempt, we provided targeted theranostic NPs, which was able to deliver both CFM-4.16 and S0456 NIR dye to the tumor site with limited off-target distribution compared to the non-targeted one. The results also showed that CD44-T-PNPs had an excellent prolonged effect in vivo confirmed by delayed renal clearance to 72 h. Probably because HA surface modification could act as a protective coating that reduces NPs opsonization and immunogenicity, leading to escape catching by RES in the blood [73,74]. The water solubility of the NPs will overcome the low solubility of anticancer agents and provide safe bio-elimination of the NPs. To the best of our knowledge, there is no FDA approved theranostic for TNBC, which is still an essential need in the clinical setup [75]. The intrinsic properties of our PNPs pave its application as a treatment option for TNBC.

## 4. Materials and Methods

### 4.1. Materials (Cell Lines and Chemicals)

TNBC cell lines MDA-MB-231 and MDA-MB-468 have been used *as* in vitro and in vivo model for human TNBC overexpressing CD44 receptors. Both cell lines were cultured in high glucose DMEM medium with 10% fetal bovine serum (FBS) and 1% penicillin-streptomycin. Cell culture was maintained at 37 °C and 5% CO_2_ conditions.

Momelotinib was purchased from Adooq Bioscience (Irvine, CA, USA). CFM-4.16 were synthesized as described before [17,76]. SMA (M = 1.6 kDa) and sodium bicarbonate were purchased from Sigma-Aldrich (St. Louis, MO, USA). Vitamin E TPGS was purchased from Antares Health Products, Inc. (Jonesborough, TN, USA). Hyaluronic acid (MW = 13 kDa) was purchased from CosChemSupply (Los Angeles, CA, USA). EDC was purchased from CovaChem (Loves Park, Illinois, USA). All the other reagents used were of analytical grade. Cell culture DMEM, FBS, penicillin-streptomycin were purchased from GIBCO (Waltham USA, MA, USA).

### 4.2. Screening of In Vitro Cell Viability (MTT Assay) and Combination Study

The cytotoxicity of CFM 4.16 and momelotinib (MMB) has been determined in both MDA-MB-231 and MDA-MB-468 at 24, 48, 72 h time points by MTT. Briefly, the cells have been plated at a density of 5 × 10^3^ cells/well in 96 well plates. After 24 h incubation, the cells were treated with different concentrations of each compound (0.390–200 μM) eight replicate/concentration. The treated plates were incubated for the indicated time points; then, the media was removed, and 100 μL of 1 mg/mL MTT was added to each well. After 4 h incubation, the MTT solution was removed, and 100 μL (DMSO) was added to solubilize the dye for 30 min with gentle shaking. The optical density (OD) of each well was measured by a plate reader at 595 nm. The cell viability (%) and the IC_50_ were calculated by GraphPad Prism (8, GraphPad, San Diego, CA, USA). IC_50_ values were calculated by plotting the log 10 nM concentrations versus cell viability % in the dose-response relationship curve:

Cell Viability (%) = (Absorbance of treated group × 100)/(Absorbances of the control group)(1)

The ability of MMB to senstize TNBC cell lines to CFM-4.16 were carried out in MDA-MB-231 and MDA-MB-468 by MTT assay. At which, five concentrations of MMB are combined with with five concentrations of CFM-4.16 based on previously determined IC50 of each drug. The cells have been treated by the noted concentrations for 72 h, followed by viability measuring by MTT assay, as previously described. The combination index (CI) and dose reduction index (DRI) were calculated by COMPUSYN software (COMPUSYN Inc, Paramus, NJ, USA). The CI is a representative quantitative measurement for the degree of drug interaction; CI < 1, CI = 1, and CI > 1 indicate synergism, additivity, and antagonism, respectively. The DRI is an indicator of how many folds the dose of each drug in a synergistic combination is reduced compared to the required dose of each drug alone to obtain the same effect.

### 4.3. Polymeric Nanoparticle Formulations

#### 4.3.1. Synthesis of the Non-Targeted (NT) SMA-TPGS and Targeted (T) HA-SMA-TPGS-Carriers

SMA-TPGS and HA-SMA-TPGS were prepared according to our previously reported method [38]. For the synthesis, 30 mg HA and 70 mg TPGS were dissolved in 50 mL deionized water (DI), then 200 mg/5 mL NaHCO3 was added to the solution. Then the pH was adjusted to 8.9, and 105 mg SMA in 10 mL DMSO was added. The reaction was left overnight until the solution becomes clear. The only difference in the NT carrier is that HA was not initially added. Both SMA-TPGS (NT carrier) and HA-SMA-TPGS (T carrier) were purified by dialysis bag (MWCO 2 kDa) for 24 h, lyophilized then characterized by FTIR and ^1^H-NMR.

#### 4.3.2. Preparation and Characterization of CFM-4.16 Loaded Polymeric NPs (PNPs)

Loading of CFM-4.16 was carried out according to our reported method [76]. First, 50 mg of carrier polymer was dissolved in 50 mL of DI water. Then 15 mg/mL of CFM-4.16 dissolved in DMSO was added to the polymer solution. Then 20 mg of EDC was added, and the pH was adjusted to 5.0, then 11 each for 30 min. Finally, the pH was adjusted to 8.0, and the free CFM-4.16 was removed by dialysis bag (MWCO 2 kDa) for 4–5 h. Eventually, the solution was lyophilized to obtain the final PNPs. The SMA-TPGS-CFM-4.16 (NT-PNPs) and HA-SMA-TPGS-CFM-4.16 (CD44-T-PNPs) morphology, hydrodynamic size, and zeta potential characterization were carried out by transmission electron microscopy (TEM, H-7500, and Hitachi Ltd., Tokyo, Japan), Dynamic Light Scattering (DLS) (Delsa Nano CTM, Beckman Coulter, Indianapolis, IN, USA) and Malvern Zetasizer (Malvern, Worcestershire, United Kingdom).

#### 4.3.3. Loading Capacity (LC%) and Encapsulation Efficiency (EE%)

The LC% and EE% of NT-PNPs and CD44-T-PNPs were measured by high-performance liquid chromatography (HPLC). The mobile phase consisted of acetonitrile 65%, methanol 20%, and 10 mM potassium dihydrogen phosphate (KH_2_PO_4_) with (pH 2) 15%, and the readout wavelength was 309 nm. Briefly, 1 mg of each formulation/1ml DI water was prepared, then 100 μg, 50 μg, and 25 μg concentrations were prepared using the mobile phase as a diluent. The average of triplicate injections of each sample was used on the standard curve equation; then, the LC%, EE%, and yield were calculated as follows:

Loading Capacity (LC%) = (Amount of CFM4.16 entrapped in the PNPs)/(Total weight of PNPs) × 100(2)

Encapsulation Efficiency (EE%) = (Amount of CFM4.16 entrapped in PNPs)/(Amount of CFM4.16 added) ×100(3)

Yield%= (Total weight of PNPs)/(Weight of used (polymer+CFM4.16) × 100(4)

#### 4.3.4. Cellular Uptake of and In Vitro Cytotoxicity PNPs

Rhodamine B, tracer dye, was loaded to NT and T carriers, in the same way, mentioned in Section 4.3.2, to form (NT-PNPs-Rhod and CD44-T-PNPs-Rhod). Their LC% was measured by UV- spectrophotometer (2910, Mettler-Toledo, LLC, Columbus, OH, USA) adjusted on 543 nm and calculated with the previously described equations in Section 4.3.3. In vitro cell uptake study was carried on MDA-MB-231 and MDA-MB-468. First, the cells were seeded in 24 well plates in a density of 1.9 × 10 ^5^/well for MDA-MB-231 and 2.4 ×10 ^5^/well for MDA-MB-468. The next day, the cells were treated with 500 nM Rhodamine B tagged PNPs for 1.5 h. The wells were then washed with PBS (3×) and fixed with 4% formalin for 15 min, followed by nuclei staining with Hoechst 33342 (1 µg/mL) for 15 min. The wells were washed and imaged by (EVOS FL Auto, Life Technologies, Waltham, MA, USA) microscope (40×) using blue and red fluorescent channels. The intensity was measured by ZEN 2012 blue edition software and significance by GraphPad Prism.

The cytotoxicity of NT-PNPs and CD44-T-PNPs were compared to their free counterpart CFM-4.16, by MTT assay in MDA-MBA-231 and MDA-MB-468 at 72 h by the previously described method (Section 4.2).

### 4.4. Combination Studies of Momelotinib (JAK/STAT inhibitor) + CFM-4.16

#### 4.4.1. In Vitro Cytotoxicity of Combinations vs. Free Drugs

The ability of PNPs to potentiate the synergistic cytotoxic effect of MMB + CFM-4.16 combination was studied by MTT assay, as previously described (Section 4.2.). Only one synergistic point in both cell lines was selected (1.5 µM momelotinib + 12.5 µM CFM-4.16). The groups were momelotinib 1.5 µM, CFM-4.16 12.5 µM, free combo (1.5 µM momelotinib + 12.5 µM CFM-4.16), NT combo (1.5 µM momelotinib + 12.5 µM NT-PNPs) and T combo (1.5 µM momelotinib + 12.5 µM CD44-T-PNPs). (Table 2).

#### 4.4.2. Morphological Alterations and Wound Healing Assay

The effect of the combination on the morphology and metastasis was studied on MDA-MB-231. The cells were plated at 90% confluence in 6 well plates and treated with the indicated concentrations of the noted compounds for the selected time points. The cells were then fixed with 70% ice-cold ethanol for 10 min and stained with 0.4% crystal violet for 1 h. The stain was poured off, and the plates were washed and dried at room temperature. The wells were photographed at 10x. The only difference in wound healing assay that each well was scratched with a sterile 200 μL micropipette tip after 24 h incubation. The wound margin was photographed by an EVOS FL Auto (Life Technologies) microscope at 10× magnification at 0, 24 , and 72 h of treatment.

#### 4.4.3. Detection of ROS Generation

ROS generation was detected using H_2_DCFDA according to the manufacturer’s instructions. Briefly, the cells were plated in 70% confluency in 6 well plates (MDA-MB-231 0.5 × 10^6^ cells/well and MDA-MB-468 1 × 10^6^ cells/well). After 24 h, the cells were treated with the indicated concentrations of the noted compounds for 12 h, and H_2_O_2_ was used as control positive. The media was then removed, wells were washed with PBS, and stained with 5 µM H_2_DCFDA for 30 min. Finally, the wells were washed with PBS and imaged by (EVOS FL Auto, Life Technologies) microscope (10×) with 10 s elapsed time. The fluorescence intensity (excitation 485 nm; emission 530 nm) was measured by ZEN 2012 blue edition software, and the difference in ROS production was calculated by GraphPad Prism [77].

#### 4.4.4. Caspase 3/7Activity Assay

Caspase 3/7 activity was measured using the Caspase-Glo^®^ 3/7 assay (Promega, Madison, WI, USA) according to the manufacturer’s recommendations. Cells were seeded in 96 well plates; the next day, cells were treated with the indicated concentrations of the noted compounds for 24 h. Then Caspase-Glo 3/7 reagent was added, and the plates were incubated for 60 min. Luminescence was measured using the microplate reader, and significance calculated by GraphPad Prism.

#### 4.4.5. Western Blot Analysis

The ability of our combination to restore the balance between the CARP-1 tumor suppressor gene and STAT3 oncogene was determined by western blot. The cells were plated in 70% confluency (1.5 × 10^6^/100 mm plate for MDA-MB-23 and 3 × 10^6^/100 mm plate for MDA-MB-468). After 24 h, the cells were treated with the indicated concentrations of the noted compounds for 12 and 48 h. The cells were harvested and lysed by RIPA buffer with protease and phosphatase inhibitor cocktail (Thermo Scientific, Waltham, MA, USA) for 15 min at 4 °C. The lysates were then centrifuged at 12,000 rpm at 4 °C for 15 min. The supernatant was collected, and the protein concentration was determined by the Protein Assay Kit (Thermo Scientific). After protein normalization, 20 µg of protein extract from each sample was separated by SDS-polyacrylamide gel electrophoresis (SDS-PAGE) 8%, followed by wet transferring to polyvinylidene difluoride (PVDF) membrane (Bio-Rad, Hercules, CA, USA) by standard procedures. The non-specific binding sites were blocked by 5% skimmed milk in 1× TBST for 1 h. Membranes were incubated with the noted dilution’s of the primary antibodies, as shown in (Table S1) overnight at 4 °C followed by incubation with 1:10,000 horseradish peroxidase-conjugated anti-rabbit secondary antibodies for 2 h at RT. The antigen-antibody complexes were detected with the ECL chemiluminescence detection system (Amersham Biosciences, Little Chalfont, Buckinghamshire United Kingdom) and exposure to X-ray film (X-Omat, Kodak, Rochester, NY, USA). The same membranes were then re-probed with anti-GAPDH antibody as an internal control.

### 4.5. Animal Studies

#### 4.5.1. Animal Husbandry and Tumor Induction

Female mice were purchased from Jackson Laboratories, housed in a sterile environment on a standard 12 h light/dark cycle, and kept on regular rodent diet and water. All animal procedures were approved by the Wayne State Animal Care and IACUC committee in accordance with NIH guidelines. Mice were subcutaneously injected in their right flanks with MDA-MB-231(5.0 × 10^6^ cells per mouse) suspended in Matrigel. Tumor growth was measured in two perpendicular directions weekly with a caliper. Tumor volumes were calculated using formula 0.5 × a × b^2^, where a is the measurement of the longest axis, and b is the other perpendicular axis. Tumors were allowed to grow for one month till the tumor became palpable with an average size of 431.5 mm^3^.

#### 4.5.2. CD44 Expression in TNBC Bearing Mice Model by Immunohistochemistry

CD44 expression was studied after obtaining tumor from TNBC bearing mice. The tumor was sectioned to 5 µm paraffin-embedded tissue sections. These sections were permeabilized with (500 µL Triton X + 25 g BSA + 500 mL PBS) 5 min, three times. Then tumor sections were blocked by 5% BSA for 1 h. The tumor area was circled by immunopen and incubated with Alexa Fluor 488 anti-mouse/human CD44 antibody (Biolegend San Diego, CA, USA) overnight in the fridge. The next day, sections were washed, and nuclei were stained with Hoechst 33342 (1 µg/mL) for 15 min. Then sections were rewashed and dried, followed by adding the mounting media and coverslips. Imaging by confocal microscope at 10× and 63× oil immersion lens adjusted for the blue channel (352–461) for Hoechst stained nuclei and green channel for Alexa fluor anti-CD44 antibody (488–519).

#### 4.5.3. TUNEL Assay

One of the critical hallmarks of late apoptosis is extensive genomic DNA fragmentation. This process generates multiple DNA double-strand breaks (DSBs) with accessible 3′-hydroxyl (3′-OH) groups that allow apoptosis detection by TUNEL assay. For this study, animals were divided into four groups; control negative, momelotinib 5 mg/kg, CFM-4.16 15 mg/kg, and T combo group received 5 mg/kg momelotinib + 15 mg/kg CD44-T-PNPs. Animals received two doses every other day, then all animals were sacrificed, and the tumor tissues were sent to the Biobank core facility for paraffin-embedded tissue sectioning for TUNEL assay. The treatment with elevated apoptosis is indicated by increased brown staining or dark-brown spots. This short-term study was carried to see the capability of the CD44-T-PNPs combo to enhance the onset of apoptosis when compared with individual drugs.

#### 4.5.4. NIR Imaging and Biodistribution Study

The feasibility of using the NT-PNPs and CD44-T-PNPs as a theranostic tool was tested in TNBC-bearing animal model. Animals were divided into three groups; free S0456 NIR dye, NT-PNPs/S0456 conjugate, and CD44-T-PNPs/S0456 conjugate. Conjugation of both formulations with S0456 NIR dye was carried as following; 10 mg of the already prepared NT-PNPs and CD44-T-PNPs were dissolved in 1:1 mixture of chloroform and methanol. Then 1 mg of S0456 NIR dye dissolved in 1 mL DI water was added to the chloroform-methanol mixture. Then chloroform and methanol were evaporated by Rotavapor (R-205, Buchi, Flawil, Switzerland). The solutions were dialyzed using a dialysis bag (MWCO 3.5 kDa) for 3 h then lyophilized. The incorporation of S0456 NIR dye to PNPs was measured by UV-spectrophotometer (Hitachi 2910) adjusted to 789 nm wavelength and calculated based on the standard curve equation. Mice were injected in the tail vein with 10 nmol/mouse of free S0456, NT-PNPs/S0456, and CD44-T-PNPs/S0456. Mice were imaged at 24 and 72 h post-injection using an In Vivo MS FX Extreme system (Carestream, San Diego, CA, USA); light source: 400 W xenon, monochrome interlined, fixed lens (10×), cooled (−60 °C), CCD camera, with 750 nm–830 nm wavelength for fluorescence, and X-ray images were captured. Both fluorescence and X-ray images of the mouse were merged to demonstrate the localization of NPs. Importantly, to understand the behavior and distribution of PNPs to tumor vs. healthy tissues, the NIR fluorescence of organ bio-distribution was carried out 72 h post-injection.

## 5. Conclusions

Despite the undeniable achievements in oncotherapy, cancers’ unsatisfactory survival rates remain an issue of concern and a challenge that scientific research is yet, to overcome. Even though chemotherapy has proven successful as a therapeutic paradigm, numerous elements deny the possibility of depending solely on it. For instance, poor solubility and off-targeting biodistribution are among the many existing challenges that limit cancer chemotherapy’s efficacy. Not only does this pr5event parenteral administration and leads to chemotherapeutics toxicity, the rapid evoking of chemotherapeutic resistance also leads to tumor relapse. Thereupon, in an attempt to address these challenges, NPs drug delivery systems (DDS) and combination strategies are currently being investigated. In this study combination of momelotinib with the CD44 directed CFM-4.16 PNPs was able to wipe out cancer cells efficiently compared to individual drugs. The developed nanomaterials’ intrinsic properties such as biodegradability, water-solubility, loading contents, and cellular internalization potentiate its application as an excellent DDS for hydrophobic CFM-4.16 and hydrophilic S0456 NIR dye. The study has opted for PNPs of smooth spherical shape, acceptable size (<100 nm), a slightly negative charge, and selective tumor uptake. Upon administration, the CD44-T-PNPs ester backbone was hydrolyzed into non-toxic products with gradual releasing of the CFM-4.16 molecules and S0456 in the tumor site with low off-target distribution, which pave its application as a promising tool for the theranostic purpose.

## Figures and Tables

**Table 1:** The loading capacity (LC%), encapsulation efficiency (EE%), and formulations yield. The CFM-4.16 LC% and EE% was determined in the NT-PNPs and CD44-T-PNPs by HPLC. Also, the formulations were weighted for yield%. Data are presented as mean ± SD, *n* = 3.

Measurements	NT-PNPs	CD44-T-PNPs
**LC%**	25.26 ± 1.22%	17 ± 1.45%
**EE%**	89.45 ± 4.89%	71 ± 6.87%
**Yield%**	81.53 ± 2.65%	95.89 ± 2.22%

**Table 2:** The selected synergistic point in MMB + CFM-4.16 combination with its CI in both MDA-MB-231 and MDA-MB-468 where MMB is momelotinib.

Combo	Dose		CI MDA-MB-231	CI MDA-MB-468
	MMB	CFM-4.16		
MMB + CFM-4.16	1.5 µM	12.5 µM	0.58	0.19

## Data Availability

The data presented in this study are available in this article (and supplementary material).

## Supplementary Materials

The following are available online at https://www.mdpi.com/2072-6694/13/4/898/s1, Figure S1: Combination Index (CI) analysis by COMPUSYN software, Figure S2: Carriers Chemical Characterization. Characterization of HA, SMA, TPGS, SMA-TPGS, and HA-SMA-TPGS by Fourier transform infrared spectroscopy (FTIR), Figure S3: Carriers Chemical Characterization. Characterization of HA, SMA, TPGS, SMA-TPGS, and HA-SMA-TPGS by proton nuclear magnetic resonance spectroscopy (1H NMR), Figure S4: Synergism underlying mechanisms of action based on our results and other supporting previously published results, Figure S5: Western blot densitometry & uncropped blots, Table S1: The molecular weights and primary antibodies dilution of the western blot tracked proteins.

## References

[1] Siegel R.L., Miller K.D., Jemal A. (2020). Cancer statistics, 2020. CA Cancer J. Clin..

[2] Tseng L.M., Hsu N.C., Chen S.C., Lu Y.S., Lin C.H., Chang D.Y., Li H., Lin Y.C., Chang H.K., Chao T.C. (2013). Distant metastasis in triple-negative breast cancer. Neoplasma.

[3] Khosravi-Shahi P., Cabezón-Gutiérrez L., Custodio-Cabello S. (2018). Metastatic triple negative breast cancer: Optimizing treatment options, new and emerging targeted therapies. Asia Pac. J. Clin. Oncol..

[4] Park J.H., Ahn J.H., Kim S.B. (2018). How shall we treat early triple-negative breast cancer (TNBC): From the current standard to upcoming immuno-molecular strategies. ESMO Open.

[5] Wahba H.A., El-Hadaad H.A. (2015). Current approaches in treatment of triple-negative breast cancer. Cancer Biol. Med..

[6] Fukuoka M., Tsuchiya R. (1994). Principles for Adjuvant and Neoadjuvant Chemotherapy. Gan To Kagaku Ryoho.

[7] Miles D., von Minckwitz G., Seidman A.D. (2002). Combination versus sequential single-agent therapy in metastatic breast cancer. Oncologist.

[8] Mokhtari R.B., Homayouni T.S., Baluch N., Morgatskaya E., Kumar S., Das B., Yeger H. (2017). Combination therapy in combating cancer. Oncotarget.

[9] O’Shea J.J., Schwartz D.M., Villarino A.V., Gadina M., McInnes I.B., Laurence A. (2015). The JAK-STAT Pathway: Impact on Human Disease and Therapeutic Intervention. Annu. Rev. Med..

[10] Leonard W.J., O′Shea J.J. (1998). JAKS AND STATS: Biological Implications. Annu. Rev. Immunol..

[11] Miklossy G., Hilliard T.S., Turkson J. (2013). Therapeutic modulators of STAT signalling for human diseases. Nat. Rev. Drug Discov..

[12] Zhao M., Gao F.H., Wang J.Y., Liu F., Yuan H.H., Zhang W.Y., Jiang B. (2011). JAK2/STAT3 signaling pathway activation mediates tumor angiogenesis by upregulation of VEGF and bFGF in non-small-cell lung cancer. Lung Cancer.

[13] Chan E., Luwor R., Burns C., Kannourakis G., Findlay J.K., Ahmed N. (2018). Momelotinib decreased cancer stem cell associated tumor burden and prolonged disease-free remission period in a mouse model of human ovarian cancer. Oncotarget.

[14] McLean J.R., Chaix D., Ohi M.D., Gould K.L. (2011). State of the APC/C: Organization, function, and structure. Crit. Rev. Biochem. Mol. Biol..

[15] Jia L., Sun Y. (2011). SCF E3 Ubiquitin Ligases as Anticancer Targets. Curr. Cancer Drug Targets.

[16] Muthu M., Somagoni J., Cheriyan V.T., Munie S., Levi E., Ashour A.E., Yassin A.E.B., Alafeefy A.M., Sochacki P., Polin L.A. (2015). Identification and Testing of Novel CARP-1 Functional Mimetic Compounds as Inhibitors of Non-Small Cell Lung and Triple Negative Breast Cancers. J. Biomed. Nanotechnol..

[17] Cheriyan V.T., Muthu M., Patel K., Sekhar S., Rajeswaran W., Larsen S.D., Polin L., Levi E., Singh M., Rishi A.K. (2016). CARP-1 functional mimetics are novel inhibitors of drug-resistant triple negative breast cancers. Oncotarget.

[18] Nabil G., Bhise K., Sau S., Atef M., El-Banna H.A., Iyer A.K. (2019). Nano-engineered delivery systems for cancer imaging and therapy: Recent advances, future direction and patent evaluation. Drug Discov. Today.

[19] Yang C., Wu T., Qi Y., Zhang Z. (2018). Recent advances in the application of vitamin E TPGS for drug delivery. Theranostics.

[20] Rodriguez V.B., Henry S.M., Hoffman A.S., Stayton P.S., Li X., Pun S.H. (2008). Encapsulation and stabilization of indocyanine green within poly(styrene-alt-maleic anhydride) block-poly(styrene) micelles for near-infrared imaging. J. Biomed. Opt..

[21] Larson N., Greish K., Bauer H., Maeda H., Ghandehari H. (2011). Synthesis and evaluation of poly(styrene-co-maleic acid) micellar nanocarriers for the delivery of tanespimycin. Int. J. Pharm..

[22] Chen C., Zhao S., Karnad A., Freeman J.W. (2018). The biology and role of CD44 in cancer progression: Therapeutic implications. J. Hematol. Oncol..

[23] Li H., Yan L., Tang E.K.Y., Zhang Z., Chen W., Liu G., Mo J. (2019). Synthesis of TPGS/Curcumin Nanoparticles by Thin-Film Hydration and Evaluation of Their Anti-Colon Cancer Efficacy In Vitro and In Vivo. Front. Pharmacol..

[24] Rakha E.A., Chan S. (2011). Metastatic Triple-negative Breast Cancer. Clin. Oncol..

[25] Hardy D., Cormier J.N., Xing Y., Liu C.C., Xia R., Du X.L. (2010). Chemotherapy-associated toxicity in a large cohort of elderly patients with non-small cell lung cancer. J. Thorac. Oncol..

[26] Wang Y., Probin V., Zhou D. (2006). Cancer therapy-induced residual bone marrow injury-Mechanisms of induction and implication for therapy. Curr. Cancer Ther. Rev..

[27] Boussios S., Pentheroudakis G., Katsanos K., Pavlidis N. (2012). Systemic treatment-induced gastrointestinal toxicity: Incidence, clinical presentation and management. Ann. Gastroenterol..

[28] Rivera E., Gomez H. (2010). Chemotherapy resistance in metastatic breast cancer: The evolving role of ixabepilone. Breast Cancer Res..

[29] Coley H.M. (2008). Mechanisms and strategies to overcome chemotherapy resistance in metastatic breast cancer. Cancer Treat. Rev..

[30] Alsaab H., Sau S., Alzhrani R. (2017). PD-1 and PD-L1 Checkpoint Signaling Inhibition for Cancer Immunotherapy: Mechanism, Combinations, and Clinical Outcome. Front. Pharmacol..

[31] Gros L., Ringsdorf H., Schupp H. (1981). Polymeric Antitumor Agents on a Molecular and on a Cellular Level?. Angew. Chemie Int. Ed. Engl..

[32] Greish K., Fang J., Inutsuka T., Nagamitsu A., Maeda H. (2003). Macromolecular Therapeutics: Advantages and Prospects with Special Emphasis on Solid Tumour Targeting. Clin. Pharmacokinet..

[33] Current Nanotechnology Treatments—National Cancer Institute. Posted August 8, 2017. https://www.cancer.gov/nano/cancer-nanotechnology/current-treatments.

[34] Suzuki F., Munakata T., Maeda H. (1988). Interferon Induction by SMANCS: A Polymer-Conjugated Derivative of Neocarzinostatin. Anticancer Res..

[35] Suzuki F., Pollard R.B., Uchimura S., Munakata T., Maeda H. (1990). Role of Natural Killer Cells and Macrophages in the Nonspecific Resistance to Tumors in Mice Stimulated With SMANCS, a Polymer-Conjugated Derivative of Neocarzinostatin. Cancer Res..

[36] Maeda H. (1994). SMANCS/Lipiodol. Gan To Kagaku Ryoho..

[37] Maeda H., Konno T., Maeda H., Edo K., Ishida N. (1997). Metamorphosis of Neocarzinostatin to SMANCS: Chemistry, Biology, Pharmacology, and Clinical Effect of the First Prototype Anticancer Polymer Therapeutic. Neocarzinostatin: The Past, Present, and Future of an Anticancer Drug.

[38] Wang Z., Sau S., Alsaab H.O., Iyer A.K. (2018). CD44 directed nanomicellar payload delivery platform for selective anticancer effect and tumor specific imaging of triple negative breast cancer. Nanomed. Nanotechnol. Biol. Med..

[39] Muthu M.S., Avinash Kulkarni S., Liu Y., Feng S.S. (2012). Development of docetaxel-loaded vitamin e TPGS micelles: Formulation optimization, effects on brain cancer cells and biodistribution in rats. Nanomedicine.

[40] Fan Z., Chen C., Pang X., Yu Z., Qi Y., Chen X., Liang H., Fang X., Sha X. (2015). Adding Vitamin E-TPGS to the Formulation of Genexol-PM: Specially Mixed Micelles Improve Drug-Loading Ability and Cytotoxicity against Multidrug-Resistant Tumors Significantly. PLoS ONE.

[41] Peer D., Karp J.M., Hong S., Farokhzad O.C., Margalit R., Langer R. (2007). Nanocarriers as an emerging platform for cancer therapy. Nat. Nanotechnol..

[42] Wickens J.M., Alsaab H.O., Kesharwani P., Bhise K., Amin M.C.I.M., Tekade R.K., Gupta U., Iyer A.K. (2017). Recent advances in hyaluronic acid-decorated nanocarriers for targeted cancer therapy. Drug Discov. Today.

[43] Necas J., Bartosikova L., Brauner P., Kolar J. (2008). Hyaluronic acid (hyaluronan): A review. Vet. Med..

[44] Ricardo S., Vieira A.F., Gerhard R., Leitão D., Pinto R., Cameselle-Teijeiro J.F., Milanezi F., Schmitt F., Paredes J. (2011). Breast cancer stem cell markers CD44, CD24 and ALDH1: Expression distribution within intrinsic molecular subtype. J. Clin. Pathol..

[45] Ma F., Li H., Wang H., Shi X., Fan Y., Ding X., Lin C., Zhan Q., Qian H., Xu B. (2014). Enriched CD44+/CD24- population drives the aggressive phenotypes presented in triple-negative breast cancer (TNBC). Cancer Lett..

[46] Kesharwani P., Banerjee S., Padhye S., Sarkar F.H., Iyer A.K. (2015). Hyaluronic Acid Engineered Nanomicelles Loaded with 3,4-Difluorobenzylidene Curcumin for Targeted Killing of CD44+ Stem-Like Pancreatic Cancer Cells. Biomacromolecules.

[47] Su Z., Chen M., Xiao Y., Sun M., Zong L., Asghar S., Dong M., Li H., Ping Q., Zhang C. (2014). ROS-triggered and regenerating anticancer nanosystem: An effective strategy to subdue tumor′s multidrug resistance. J. Control. Release.

[48] Chithrani B.D., Chan W.C.W. (2007). Elucidating the mechanism of cellular uptake and removal of protein-coated gold nanoparticles of different sizes and shapes. Nano Lett..

[49] Ganesh K., Archana D. (2013). Review Article on Targeted Polymeric Nanoparticles: An Overview. Am. J. Adv. Drug Deliv..

[50] Wang W., Gaus K., Tilley R.D., Gooding J.J. (2019). The impact of nanoparticle shape on cellular internalisation and transport: What do the different analysis methods tell us?. Mater. Horizons.

[51] Lu Y., Zhang E., Yang J., Cao Z. (2018). Strategies to improve micelle stability for drug delivery. Nano Res..

[52] Fröhlich E. (2012). The role of surface charge in cellular uptake and cytotoxicity of medical nanoparticles. Int. J. Nanomed..

[53] Chenthamara D., Subramaniam S., Ramakrishnan S.G., Krishnaswamy S., Essa M.M., Lin F.H., Qoronfleh M.W. (2019). Therapeutic efficacy of nanoparticles and routes of administration. Biomater. Res..

[54] Tsirikis P., Xiang S., Selomulya C., Plebanski M., Ficai A., Grumezescu A.M. (2017). Design of Nanoparticle Structures for Cancer Immunotherapy. Nanostructures for Cancer Therapy.

[55] Schäfer T., Noseck U., Huber F., Bouby M., Brendlé J., Büchner S., Darbha G.K., Goetz R., Flügge J., Hauser W. (2013). Colloid/Nanoparticle Formation and Mobility in the Context of Deep Geological Nuclear Waste Disposal (Project KOLLORADO-2; Final Report).

[56] Blanco E., Shen H., Ferrari M. (2015). Principles of nanoparticle design for overcoming biological barriers to drug delivery. Nat. Biotechnol..

[57] Bose T., Latawiec D., Mondal P.P., Mandal S. (2014). Overview of nano-drugs characteristics for clinical application: The journey from the entry to the exit point. J. Nanoparticle Res..

[58] Nag O.K., Delehanty J.B. (2019). Active cellular and subcellular targeting of nanoparticles for drug delivery. Pharmaceutics.

[59] Honary S., Zahir F. (2013). Effect of Zeta Potential on the Properties of Nano-Drug Delivery Systems-A Review (Part 2). Trop. J. Pharm. Res..

[60] McConnell K.I., Shamsudeen S., Meraz I.M., Mahadevan T.S., Ziemys A., Rees P., Summers H.D., Serda R.E. (2016). Reduced Cationic Nanoparticle Cytotoxicity Based on Serum Masking of Surface Potential. J. Biomed. Nanotechnol..

[61] Jokerst J.V., Lobovkina T., Zare R.N., Gambhir S.S. (2011). Nanoparticle PEGylation for imaging and therapy. Nanomedicine.

[62] Çalış S., Öztürk Atar K., Arslan F.B., Eroğlu H., Çapan Y., Mohapatra S., Ranjan S., Dasgupta N., Kumar R., Thomas S. (2019). Nanopharmaceuticals as Drug-Delivery Systems: For, Against, and Current Applications. Nanocarriers for Drug Delivery.

[63] Dong H., Pang L., Cong H., Shen Y., Yu B. (2019). Application and design of esterase-responsive nanoparticles for cancer therapy. Drug Deliv..

[64] Pawar A., Prabhu P. (2019). Nanosoldiers: A promising strategy to combat triple negative breast cancer. Biomed. Pharmacother..

[65] Thomas S.J., Snowden J.A., Zeidler M.P., Danson S.J. (2015). The role of JAK/STAT signalling in the pathogenesis, prognosis and treatment of solid tumours. Br. J. Cancer.

[66] Nascimento A.S., Peres L.L., Faria A.V.S., Milani R., Silva R.A., da Costa Fernandes C.J., Peppelenbosch M.P., Ferreira-Halder C.V., Zambuzzi W.F. (2017). Phosphoproteome profiling reveals critical role of JAK-STAT signaling in maintaining chemoresistance in breast cancer. Oncotarget.

[67] Marotta L.L.C., Almendro V., Marusyk A., Shipitsin M., Schemme J., Walker S.R., Bloushtain-Qimron N., Kim J.J., Choudhury S.A., Maruyama R. (2011). The JAK2/STAT3 signaling pathway is required for growth of CD44 +CD24- stem cell-like breast cancer cells in human tumors. J. Clin. Invest..

[68] Lu L., Dong J., Wang L., Xia Q., Zhang D., Kim H., Yin T., Fan S., Shen Q. (2018). Activation of STAT3 and Bcl-2 and reduction of reactive oxygen species (ROS) promote radioresistance in breast cancer and overcome of radioresistance with niclosamide. Oncogene.

[69] Choi S.M., Kim D.H., Chun K.S., Choi J.S. (2019). Carnosol induces apoptotic cell death through ROS-dependent inactivation of STAT3 in human melanoma G361 cells. Appl. Biol. Chem..

[70] Cai W., Yang X., Han S., Guo H., Zheng Z., Wang H., Guan H., Jia Y., Gao J., Yang T. (2015). Notch1 Pathway Protects against Burn-Induced Myocardial Injury by Repressing Reactive Oxygen Species Production through JAK2/STAT3 Signaling. Oxid. Med. Cell. Longev..

[71] Zou Z., Chang H., Li H., Wang S. (2017). Induction of reactive oxygen species: An emerging approach for cancer therapy. Apoptosis.

[72] Brentnall M., Rodriguez-Menocal L., De Guevara R.L., Cepero E., Boise L.H. (2013). Caspase-9, caspase-3 and caspase-7 have distinct roles during intrinsic apoptosis. BMC Cell Biol..

[73] Li W., Zhou C., Fu Y., Chen T., Liu X., Zhang Z., Gong T. (2020). Targeted delivery of hyaluronic acid nanomicelles to hepatic stellate cells in hepatic fibrosis rats. Acta Pharm. Sin. B.

[74] Almalik A., Benabdelkamel H., Masood A., Alanazi I.O., Alradwan I., Majrashi M.A., Alfadda A.A., Alghamdi W.M., Alrabiah H., Tirelli N. (2017). Hyaluronic Acid Coated Chitosan Nanoparticles Reduced the Immunogenicity of the Formed Protein Corona. Sci. Rep..

[75] Thakur V., Kutty R.V. (2019). Recent advances in nanotheranostics for triple negative breast cancer treatment. J. Exp. Clin. Cancer Res..

[76] Cheriyan V.T., Alsaab H.O., Sekhar S., Stieber C., Kesharwani P., Sau S., Muthu M., Polin L.A., Levi E., Iyer A.K. (2017). A CARP-1 functional mimetic loaded vitamin E-TPGS micellar nano-formulation for inhibition of renal cell carcinoma. Oncotarget.

[77] Zaidieh T., Smith J.R., Ball K.E., An Q. (2019). ROS as a novel indicator to predict anticancer drug efficacy. BMC Cancer.

