# Supplementary material for: CD44 Targeted Nanomaterials for Treatment of Triple-Negative Breast Cancer
Source: Cancers (Basel). 2021 Feb 20;13(4):898. doi: 10.3390/cancers13040898 (PMC7924562; doi:10.3390/cancers13040898)
Supplement: Supplementary file 1 [file cancers-13-00898-s001.pdf]

Supplementary Materials: CD44 Targeted Nanomaterials for Treatment of Triple-Negative Breast Cancer

Ghazal Nabil, Rami Alzhrani, Hashem O. Alsaab, Mohammed Atef, Samaresh Sau, Arun K. Iyer and Hossny El Banna

MDA-MB-231		CFM-4.16				
		0.7	1.5	3.12	6.25	12.5
Momelotinib	0.7	0.31	0.44	0.87	1.71	0.24
	1.5	0.91	1.39	3.44	5.2	0.58
	3.12	1.21	1	1.63	1.65	0.25
	6.25	0.86	0.88	1.41	1.94	0.38
	12.5	0.99	1.11	1.1	1.33	0.52

MDA-MB-468		CFM-4.16				
		0.7	1.5	3.12	6.25	12.5
Momelotinib	0.7	0.38	0.98	2.37	7.28	0.2
	1.5	0.7	1.04	1.9	2.71	0.19
	3.12	0.7	0.84	0.94	1.51	0.31
	6.25	1.02	1.17	1.29	1.41	0.56
	12.5	1.55	1.64	1.6	1.59	1.04

< 0.9 Synergism

0.9 -1.1 Additive

> 1.1 Antagonism

Figure S1. Combination Index (CI) analysis by COMPUSYN software. The tables show the CI values of the 25 points of momelotinib +CFM-4.16 combination in both MDA-MB-231 and MDA-MB-468.

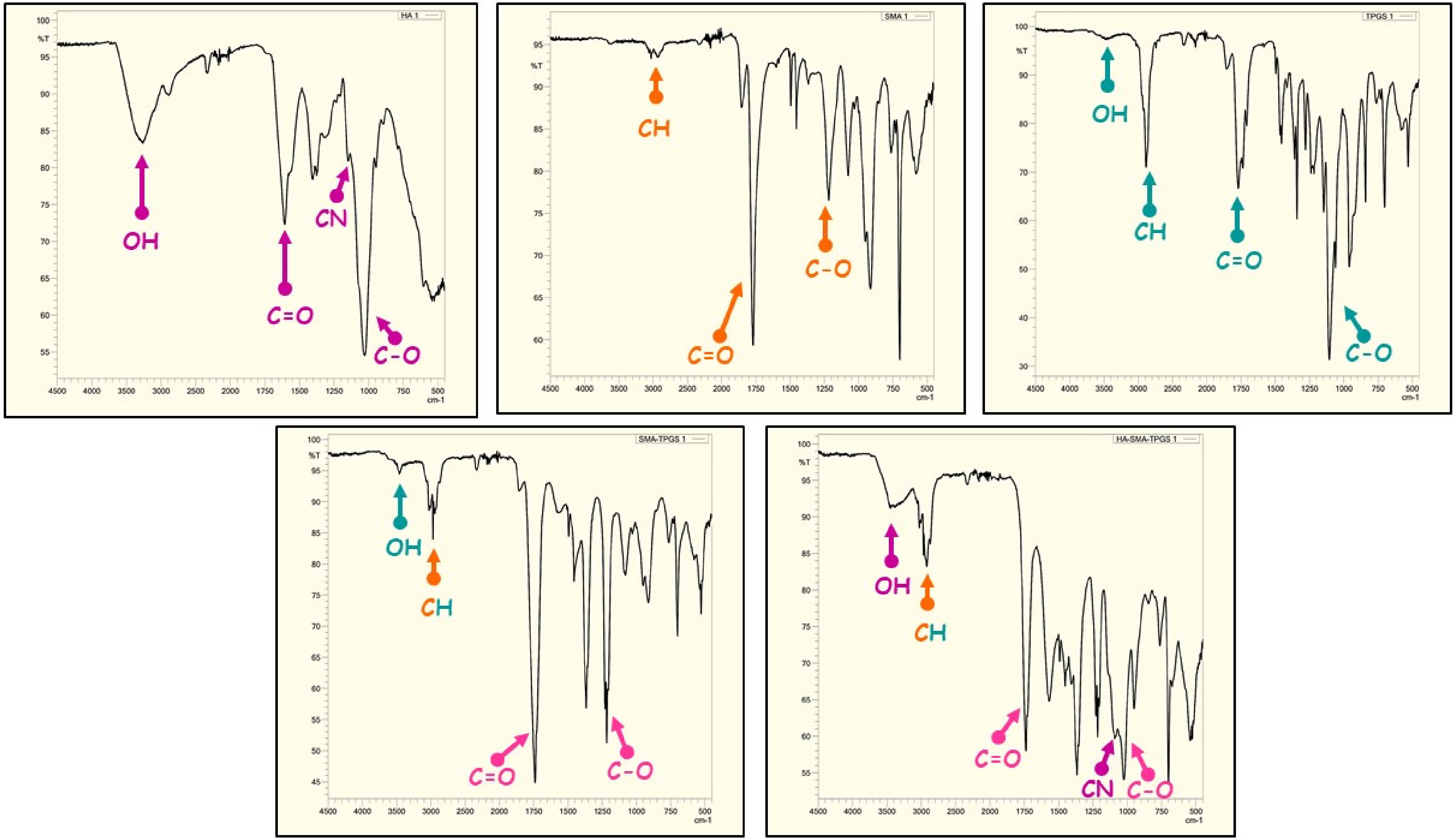

Figure S2. Carriers Chemical Characterization. Characterization of HA, SMA, TPGS, SMA-TPGS, and HA-SMA-TPGS by Fourier transform infrared spectroscopy (FTIR).

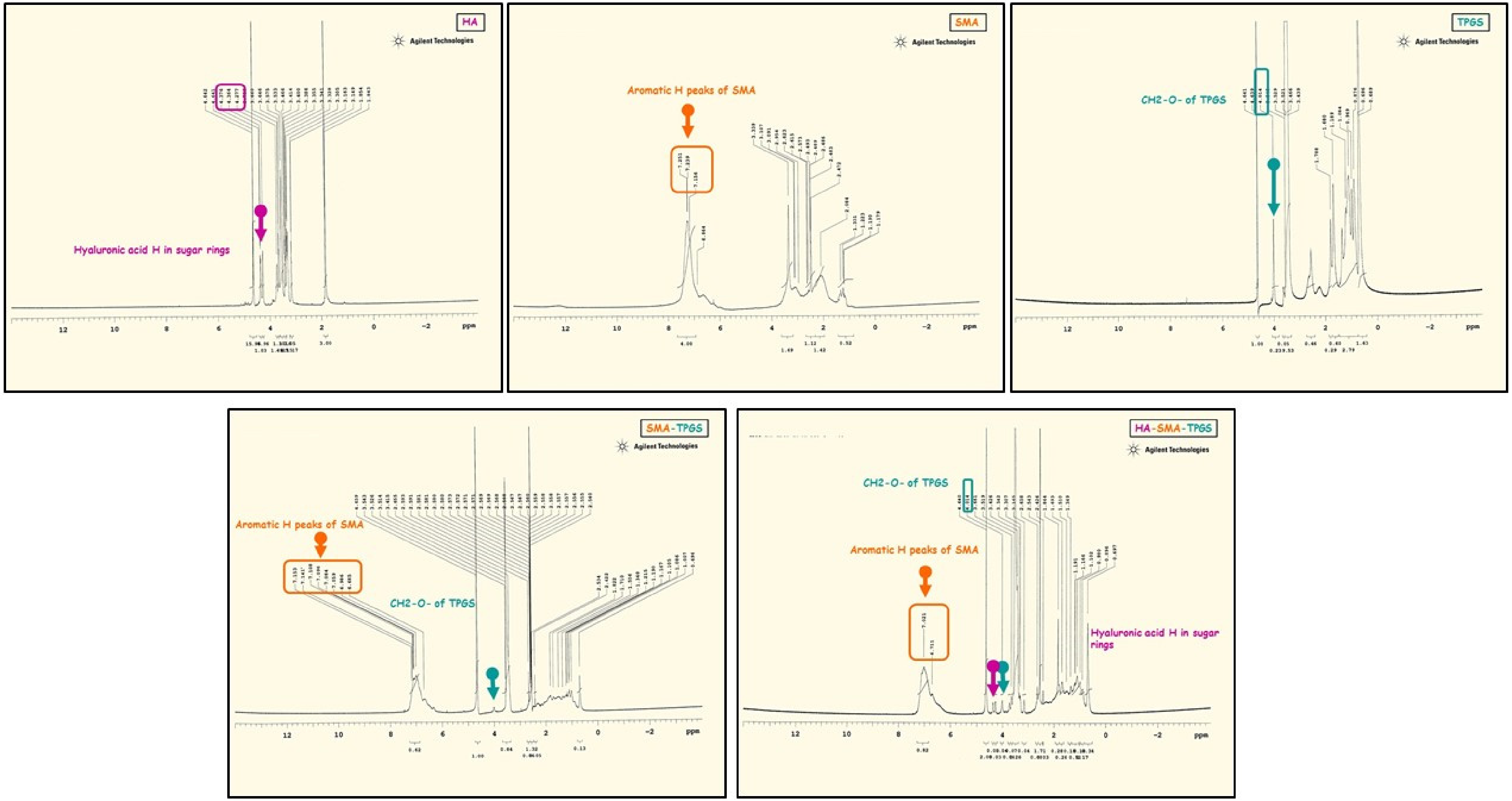

Figure S3. Carriers Chemical Characterization. Characterization of HA, SMA, TPGS, SMA-TPGS, and HA-SMA-TPGS by proton nuclear magnetic resonance spectroscopy (^{1}H NMR).

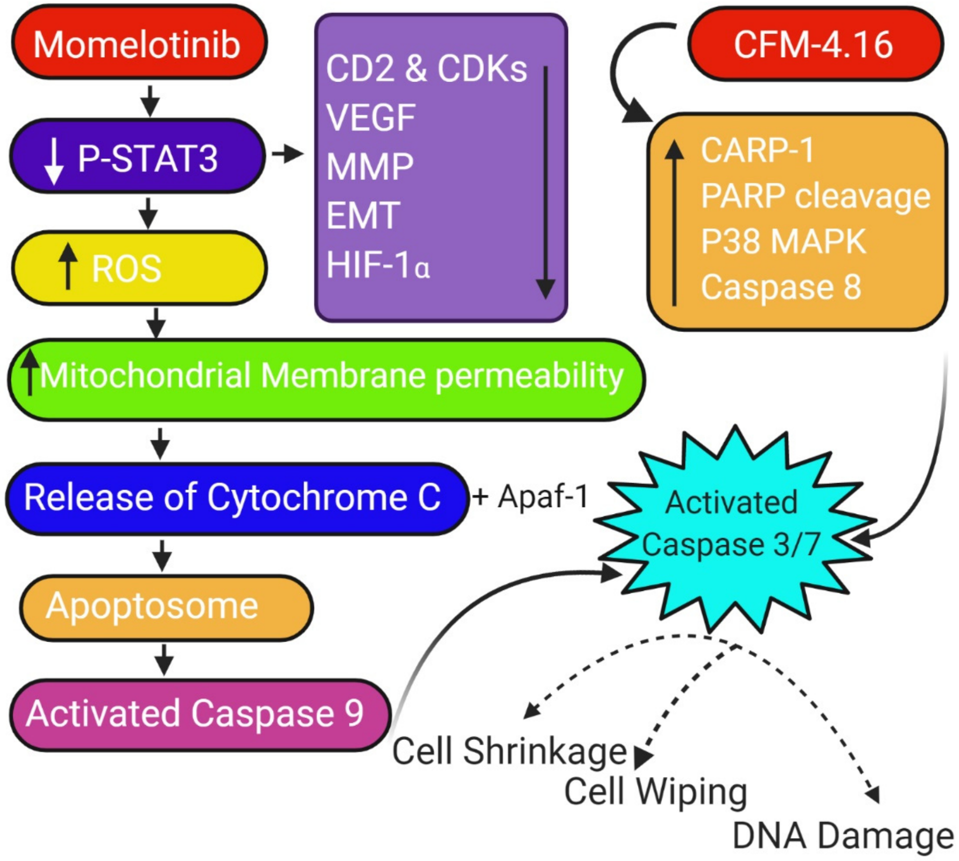

Figure S4. Synergism underlying mechanisms of action based on our results and other supporting previously published results [1–10] Treating the cells with Momelotinib + CFM-4.16 combination evokes cascading events that end up by activating caspase 3/7, the irreversible executer of cellular apoptosis.

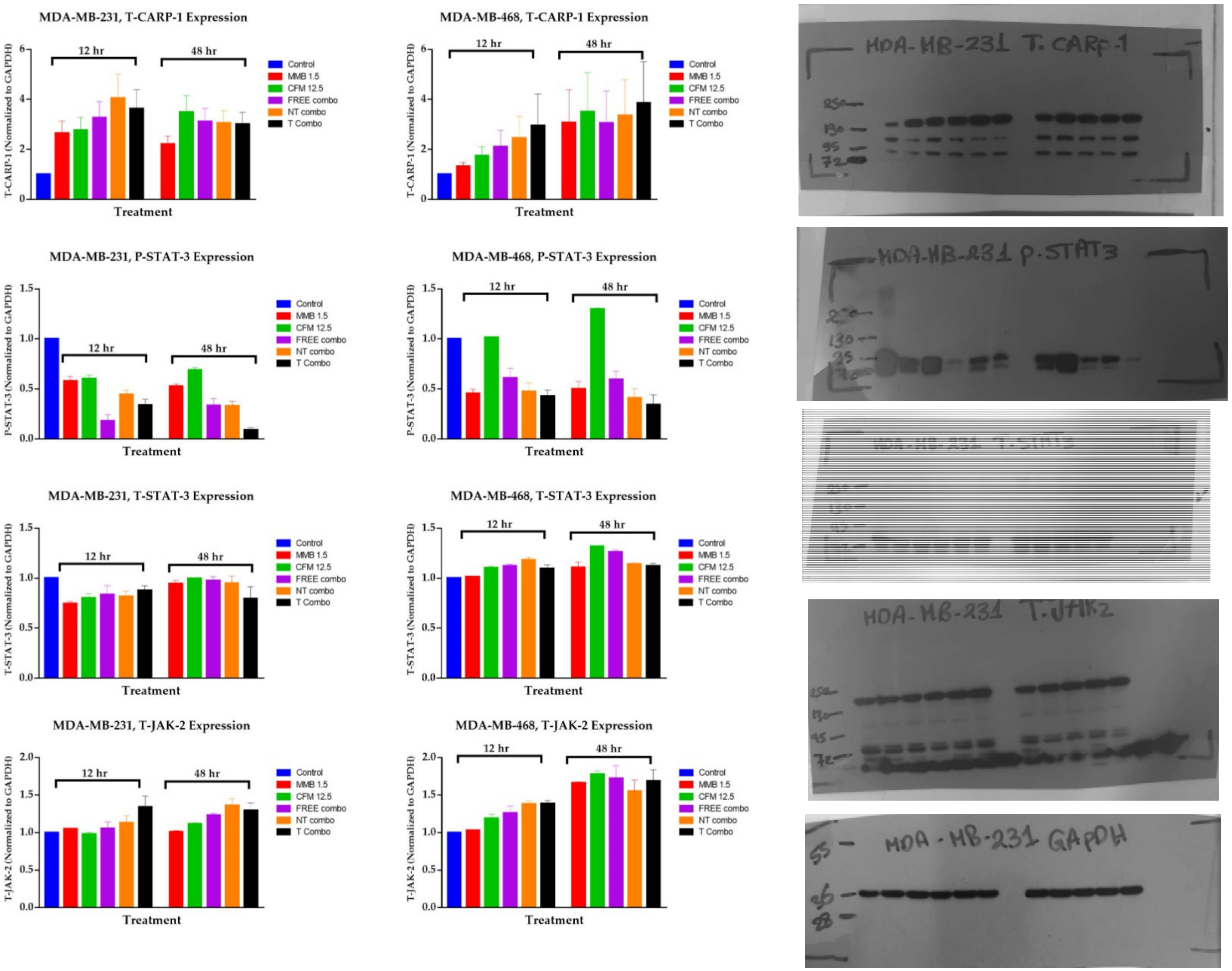

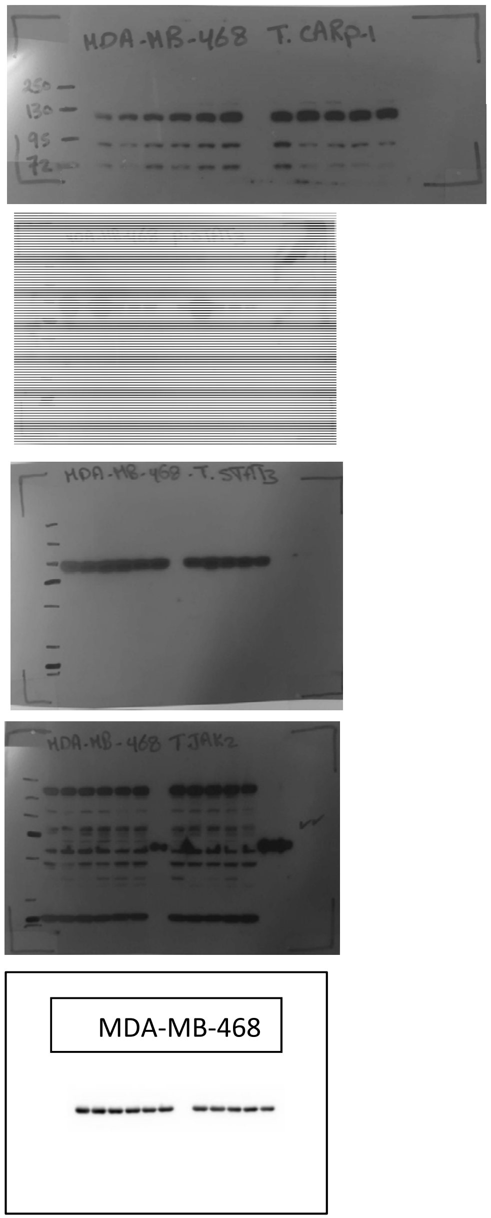

Figure S5. Western blot densitometry & uncropped blots.

Table S1. The molecular weights and primary antibodies dilution of the western blot tracked proteins.

Protein	MW kDa	Primary Antibody	Source
Total CARP-1	150	1:2000	Dr.Arun Rishi
Total STAT3	88	1:1000	Proteintech
Phosphorylated STAT3	88	1:1000	Dr.Arun Rishi
Total JAK2	150	1:1000	Proteintech
GAPDH	37	1:45000	Abcam

Reference

1. Brentnall, M., Rodriguez-Menocal, L., De Guevara, R.L., Cepero, E., Boise, L.H. Caspase-9, caspase-3 and caspase-7 have distinct roles during intrinsic apoptosis. *BMC Cell Biol.* **2013**, *14*, 32, doi:10.1186/1471-2121-14-32.
2. Cai, W., Yang, X., Han, S., Guo, H., Zheng, Z., Wang, H., Guan, H., Jia, Y., Gao, J., Yang, T.; et al. Notch1 Pathway Protects against Burn-Induced Myocardial Injury by Repressing Reactive Oxygen Species Production through JAK2/STAT3 Signaling. *Oxid. Med. Cell. Longev.* **2016**, 5638943, doi:10.1155/2016/5638943.
3. Chan, E., Luwor, R., Burns, C., Kannourakis, G., Findlay, J.K., Ahmed, N. Momelotinib decreased cancer stem cell associated tumor burden and prolonged disease-free remission period in a mouse model of human ovarian cancer. *Oncotarget* **2018**, *9*, 16599–16618, doi: 10.18632/oncotarget.24615.
4. Cheriyan, V.T., Muthu, M., Patel, K., Sekhar, S., Rajeswaran, W., Larsen, S.D., Polin, L., Levi, E., Singh, M., Rishi, A.K. CARP-1 functional mimetics are novel inhibitors of drug-resistant triple negative breast cancers. *Oncotarget* **2016**, *7*, 73370–73388, doi:10.18632/oncotarget.12333.
5. Choi, S.M., Kim, D.H., Chun, K.S., Choi, J.S. Carnosol induces apoptotic cell death through ROS-dependent inactivation of STAT3 in human melanoma G361 cells. *Appl. Biol. Chem.* **2019**, *62*, 1–11, doi:10.1186/s13765-019-0463-z.
6. Lu, L., Dong, J., Wang, L., Xia, Q., Zhang, D., Kim, H., Yin, T., Fan, S., Shen, Q. Activation of STAT3 and Bcl-2 and reduction of reactive oxygen species (ROS) promote radioresistance in breast cancer and overcome of radioresistance with niclosamide. *Oncogene* **2018**, *37*, 5292–5304, doi:10.1038/s41388-018-0340-y.
7. Marotta, L.L.C., Almendro, V., Marusyk, A., Shipitsin, M., Schemme, J., Walker, S.R., Bloushtain-Qimron, N., Kim, J.J., Choudhury, S.A., Maruyama, R.; et al. The JAK2/STAT3 signaling pathway is required for growth of CD44 +CD24- stem cell-like breast cancer cells in human tumors. *J. Clin. Invest.* **2011**, *121*, 2723–2735, doi:10.1172/JCI44745.
8. Muthu, M., Somagoni, J., Cheriyan, V.T., Munie, S., Levi, E., Ashour, A.E., Yassin, A.E.B., Alafeefy, A.M., Sochacki, P., Polin, L.A.; et al. Identification and Testing of Novel CARP-1 Functional Mimetic Compounds as Inhibitors of Non-Small Cell Lung and Triple Negative Breast Cancers. *J. Biomed. Nanotechnol.* **2015**, *11*, 1608–1627, doi:10.1166/jbn.2015.2099.
9. Thomas, S.J., Snowden, J.A., Zeidler, M.P., Danson, S.J. The role of JAK/STAT signalling in the pathogenesis, prognosis and treatment of solid tumours. *Br. J. Cancer* **2015**, *113*, 365–371, doi: 10.1038/bjc.2015.233.
10. Zou, Z., Chang, H., Li, H., Wang, S. Induction of reactive oxygen species: An emerging approach for cancer therapy. *Apoptosis* **2017**, *22*, 1321–1335, doi:10.1007/s10495-017-1424-9.

.
.
